# Dendritic cell-natural killer cell cross-talk modulates T cell activation in response to influenza A viral infection

**DOI:** 10.3389/fimmu.2022.1006998

**Published:** 2022-12-22

**Authors:** Abigail G. Harvey, Athens M. Graves, Chandana K. Uppalapati, Saoirse M. Matthews, Stephanie Rosenberg, Emma G. Parent, Madison H. Fagerlie, Jack Guinan, Brina S. Lopez, Lisa M. Kronstad

**Affiliations:** ^1^ Master of Biomedical Sciences Program, Midwestern University, Glendale, AZ, United States; ^2^ Department of Microbiology and Immunology, College of Graduate Studies, Midwestern University, Glendale, AZ, United States; ^3^ Arizona College of Osteopathic Medicine, Midwestern University, Glendale, AZ, United States; ^4^ Farm Animal Medicine, College of Veterinary Medicine, Midwestern University, Glendale, AZ, United States

**Keywords:** dendritic cells, natural killer cells, T cells, influenza, pandemic, H1N1, H3N2, cross-talk

## Abstract

Influenza viruses lead to substantial morbidity and mortality including ~3-5 million cases of severe illness and ~290,000-650,000 deaths annually. One of the major hurdles regarding influenza vaccine efficacy is generating a durable, robust cellular immune response. Appropriate stimulation of the innate immune system is key to generating cellular immunity. Cross-talk between innate dendritic cells (DC) and natural killer (NK) cells plays a key role in activating virus-specific T cells, yet the mechanisms used by influenza A viruses (IAV) to govern this process remain incompletely understood. Here, we used an *ex vivo* autologous human primary immune cell culture system to evaluate the impact of DC-NK cell cross-talk and subsequent naïve T cell activation at steady-state and after exposure to genetically distinct IAV strains–A/California/07/2009 (H1N1) and A/Victoria/361/2011 (H3N2). Using flow cytometry, we found that exposure of DCs to IAV in co-culture with NK cells led to a decreased frequency of CD83^+^ and CD86^+^ cells on DCs and an increased frequency of HLA-DR^+^ on both DCs and NK cells. We then assessed the outcome of DC-NK cell cross-talk on T cell activation. At steady-state, DC-NK cell cross-talk increased pan T cell CD69 and CD25 expression while exposure to either IAV strain reduced pan T cell CD25 expression and suppressed CD4^+^ and CD8^+^ T cell IFN-γ and TNF production, following chemical stimulation with PMA/Ionomycin. Moreover, exposure to A/Victoria/361/2011 elicited lower IFN-γ production by CD4^+^ and CD8^+^ T cells compared with A/California/07/2009. Overall, our results indicate a role for DC-NK cell cross-talk in T cell priming in the context of influenza infection, informing the immunological mechanisms that could be manipulated for the next generation of influenza vaccines or immunotherapeutics.

## Introduction

Influenza viruses cause annual seasonal epidemics and sporadic pandemics of zoonotic origin. The World Health Organization estimates the annual global burden of influenza at ~1 billion cases, including ~290,000-650,000 deaths. The U.S. economic burden of influenza-related illness has been estimated to reach $87 billion annually (1, 2). The capacity of influenza viruses for rapid evolution *via* genetic reassortment and antigenic drift leads to the evasion of immune surveillance by host antibodies specific to viral surface antigens. CD8^+^ T cells recognize viral peptides derived from internal viral components that are less subject to antibody-selected antigenic drift, and therefore more conserved across influenza strains and subtypes (3, 4). Indeed, CD8^+^ T cells specific to conserved viral epitopes correlate with protection against symptomatic influenza illness, making a vaccine capable of stimulating heterologous CD8^+^ T cells an attractive target in vaccine design (5–9).

Initiating antigen-specific CD8^+^ T cell responses relies partly on dendritic cells (DC), which display cytosolic antigen on major histocompatibility (MHC) class I molecules *via* cross-presentation. Cross-presentation is crucial in activating effective adaptive immune responses to pathogens that do not typically infect DCs directly (10). DCs also shape the nature of the ensuing adaptive immune response depending on their specific phenotypic and functional state–inducing adaptive immune responses ranging from tolerogenic to stimulatory including polarizing the immune response towards T_H_1, T_H_2, or T_H_17 (11, 12). The ability to engineer a desired adaptive immune response by exploiting the plasticity of DCs has made them a prime target in vaccine development. However, few studies have examined the possibility of DCs as an approach for universal influenza viral vaccine development, despite some promising studies demonstrating their success when used in populations most at risk for developing severe influenza-related illnesses such as immunocompromised individuals (13).

DC-based vaccines have been largely explored for cancer immunotherapy and less frequently, for the prevention of infectious diseases (14, 15). While pre-clinical testing of DC vaccines has frequently shown promising results, clinical trials have faced challenges including induction of an effective activation state (16–21). Recent studies have highlighted the potential of employing natural killer (NK) cells to improve the potency of DC vaccine preparations (22, 23). Bidirectional interactions between DCs and NK cells (cross-talk) include the formation of conjugates and influence both the magnitude and quality of the innate and adaptive immune responses (22, 24–26). DCs act on NK cells to augment their cytotoxic potential, while NK cells, in turn, may act to counteract tumor or viral immune strategies by eliminating infected DCs with impaired antigen presentation functions. This cooperative interaction is mediated both by receptor-ligand interactions and by soluble mediators. For example, ligation of the NK cell activation receptor NKp30 and tumor necrosis factor (TNF; formerly TNF-α) have both been shown to be involved in NK cell-mediated maturation of DCs (26, 27). Because DCs possess extensive functional plasticity, the outcome of DC-NK cell cross-talk has the potential to favor the development of DC-mediated CD4^+^ T_H_1 polarized response to antigens cross-presented by bystander DCs, which is responsible for protective immunity against intracellular pathogens (28, 29). Indeed, NK cell depletion and reconstitution experiments showed that NK cell secretion of interferon (IFN)-γ was necessary for the activation and expansion of T_H_1 CD4^+^ T cells following DC vaccination (30). In mice, Martín-Fontecha et al. showed that NK cells were rapidly recruited to secondary lymphoid organs–the location of DC-mediated naïve T cell stimulation–after injection of the animals with lipopolysaccharide-matured DCs (30). This trafficking occurred in a manner dependent on the chemokine receptor CXCR3 (30). In humans, Draghi et al. found that influenza-infected DCs enhanced both NK cell expression of CD69, cytolytic activity, and IFN-γ production (31). Despite this knowledge, studies investigating the cellular changes that occur on T cells due to DC-NK cell cross-talk after influenza exposure are lacking.

In this study, we used an *ex vivo* autologous human cell triple co-culture system to test the hypothesis that DC-NK cell cross-talk at varying cell ratios may influence the phenotypic and functional profile of T cell and that the degree of T cell activation may differ following exposure of DCs to the A/California/07/2009 (H1N1) (Cal/09) strain compared with A/Victoria/361/2011 (H3N2) (Vic/11) seasonal strain. These strains were selected based on a prior report that demonstrated that NK cells displayed a heightened IFN-γ response to Cal/09 compared to other influenza A viral strains tested, including Vic/11 (32). We found that influenza A virus (IAV) influences the expression of co-stimulatory (CD83 and CD86) and antigen presentation (HLA-DR) molecules on DCs cultured with NK cells. Further DC-NK cell cross-talk influenced the cytokine milieu and heightened the expression of T cell surface activation markers, yet this T cell activation profile was curtailed by IAV, including a strain-specific reduction in T cell IFN-γ production.

## Materials and methods

### Virus production and titration

A/Victoria/361/2011 (Vic/11; H3N2) (BEI Resources) and A/California/07/2009 (Cal/09: H1N1) (BEI Resources) were propagated in 10-day-old specific pathogen-free embryonated chicken eggs (Charles River Laboratories Wilmington, MA) at 35˚C and with 55-65% humidity. Allantoic fluid was harvested at 48 HPI followed by overnight incubation at 4˚C. Each of the Vic/11 and Cal/09 batches was grown from a seed stock and multiple batches were tested. Infectious influenza titer was determined by a standard plaque assay on MDCK cells in the presence of 2 µg/mL L-1-tosylamide-2-phenylethyl chloromethyl ketone (TPCK)-treated trypsin. IAV inactivation was performed by delivering 2400 µJ/cm^2^ of UV light (254 nm) using a Strata linker 1800 (Stratagene, La Jolla, CA.). Virus inactivation was verified by plaque assay or by intracellular influenza nucleoprotein antibody staining followed by analysis by flow cytometry.

### Peripheral blood mononuclear cell isolation

Whole blood (~350 mL) was collected from both male and female healthy adult donors *via* antecubital venipuncture into BD vacutainer, and Sodium Heparin blood collection tubes (BD, Franklin Lakes, NJ). Blood was diluted 2-fold in calcium- and magnesium-free phosphate-buffered saline (PBS pH: 7.2; Fisher Scientific, ​​Waltham, MA) within 30 min of collection. A single-step density gradient centrifugation was performed to isolate peripheral blood mononuclear cells (PBMCs) by slowly layering diluted blood (30 mL) over 15 mL of Lymphoprep™ (STEMCELL Technologies, Vancouver, Canada) contained within either a 50 mL SepMate™ (STEMCELL Technologies) or a 50 mL conical tube. Stepwise centrifugation at 1,200 x *g* for 10 min at room temperature with slow deceleration was performed. The buffy coat interface containing PBMCs was collected into 50 mL conical tubes and pelleted *via* centrifugation (1,400 RPM for 10 min). Residual red blood cells were removed with treatment with ACK Lysing Buffer (Gibco, Waltham, MA). PBMCs were then washed twice with 1X PBS, pelleted as above, and cryopreserved at -80°C in 90% (v/v) fetal bovine serum (FBS; R&D Systems, Minneapolis, MN) and 10% (v/v) dimethyl sulfoxide (Corning, Corning, NY) in cryovials labeled with each donor’s unique sample identification number, followed by transfer to liquid nitrogen storage within 24 h.

### Differentiation of monocytes into monocyte-derived dendritic cells

**​​**Human MoDCs were generated using established protocols (33). Briefly, untouched monocytes were purified from the PBMC population by negative selection using magnetic-activated cell sorting (MACS^®^) with the Pan Monocyte Isolation kit (Miltenyi Biotec, Auburn, CA, CAT#130-096-537). Monocytes were then seeded at a density of 0.5 – 1.0 x 10^6^ cells/well. The monocyte population was further enriched by incubating cells in serum-free RPMI 1640 medium (Fisher Scientific, ​​Waltham, MA) at 37°C and 5% CO_2_ for 2 h in 12-well Nunclon™ Delta surface-treated flat bottom plates (Thermo Fisher Scientific, Waltham, MA). Non-adherent cells were removed by gently washing the cells with pre-warmed culture medium. Adherent monocytes were then cultured with RPMI 1640 supplemented with 10% FBS, 1% L-Glutamine, and 1% Penicillin/Streptomycin (Thermo Fisher Scientific, Waltham, MA), recombinant human granulocyte-macrophage colony-stimulating factor (rhGM-CSF) (800 U/L; Thermo Fisher Scientific, Waltham, MA) and recombinant human interleukin 4 (rhIL-4) (500 U/L; Thermo Fisher Scientific, Waltham, MA) to induce MoDC differentiation. Media was changed on day two and day five of culture by removing the top half of the medium and replacing it with equal volumes of fresh RP10 medium supplemented with rhGM-CSF and rhIL-4.

### Cell purification, cell stimulation, and IAV infection

For each experiment, cryopreserved PBMCs were thawed and washed in RP10 medium (RPMI 1640 medium supplemented with 10% FBS plus 2 mM L-glutamine, 100 U/ml penicillin, and 100 U/ml streptomycin). Autologous, untouched naïve T cells and NK cells were purified by negative selection using MACS according to the manufacturer’s instructions. Briefly, T cells were purified using the human Naïve Pan T Cell Isolation Kit (Miltenyi Biotec; CAT# 130-097-095). This kit depletes PBMCs of cells other than naïve T cells using antibodies specific to CD45RO, CD14, CD15, CD16, CD19, CD25, CD34, CD36, CD57, CD123, HLA-DR, CD235a, and CD244 yielding cells that are CD3^+^CD45RA^+^CD197^+^. NK cells were purified using the human NK Cell Isolation Kit (Miltenyi Biotec; CAT#130-092-657). The viability for each cell type was determined using a micro flow cytometer with propidium iodide (Moxi Flow; ORFLO Technologies, Ketchum, ID) or a hemocytometer. MoDCs were pelleted and re-suspended in serum-free RPMI, then plated into wells of a 96-well U-bottom tissue culture treated plate (CELLTREAT, Pepperell, MA) at a seeding density of 2 X 10^5^ cells/200uL/well and 0.5 X 10^5^cells/50uL/well. Cal/09 or Vic/11 was then adsorbed on MoDCs at a multiplicity of infection (MOI) of 3 for 1 h at 37°C and 5% CO_2_ in FBS-free RPMI. At 1 HPI, virus-containing supernatant was decanted, and MoDCs were either co-cultured with NK cells (1:4 or 4:1 ratios) and/or NK cells and pan naïve T cells (1:1:1, 4:1:1, or 1:4:1 ratios) in serum-containing RP10 medium–which acts to further inhibit viral entry–for the indicated time point (34). NK cells or T cells were stimulated with 162 nM Phorbol-Myristate-Acetate and 2.68 μM ionomycin (PMA/I) for 6 h (NK cells) or 12 h (T cells) for surface marker activation or 4 h for T cell intracellular cytokine staining. MoDCs were stimulated with 30 µg/mL Poly (I:C) for 24 h (*In vivo*gen). In transwell experiments, MoDCs and NK cells were seeded in a 24-well plate and separated during culture by a semipermeable membrane of 0.4-μm pore size (Corning).

### Flow cytometry

Cell purity after magnetic isolation was accessed using indicated lineage markers and analytical flow cytometry. For MoDCs and NK cell co-culture experiments, cells were first stained with LIVE/DEAD™ Fixable Violet Dead Cell Stain kit (Thermo Fisher Scientific), followed by surface staining with APC anti-human CD7 (clone: CD7-6B7; BioLegend^®^), Phycoerythrin (PE)-Cy7 anti-human CD56 (clone: B159; BD Biosciences), PE anti-human CD83 (clone: HB15e; BioLegend^®^, San Diego, CA), Brilliant Violet 510™ anti-human CD86 (clone: IT2.2; BioLegend^®^), Brilliant Violet 570™ anti-human HLA-DR (clone: L243; BioLegend^®^), and PE anti-human HLA-A, B, C (clone: W6/32; BioLegend^®^) antibodies. For MoDC, NK cell, and T cell co-culture experiments, cells were first stained with Live-or-Dye™ fixable AmCyan (Biotium, Fremont, CA) followed by staining with PE anti-human CD3-phycoerythrin (clone: UCHT1; BioLegend^®^), Pacific Blue™ anti-human CD69 (clone: FN50; BioLegend^®^), and anti-CD25-Alexa-647 (clone: BC96; BioLegend^®^) antibodies. Fixation and permeabilization with FACS Lyse and FACS Perm II (BD Pharmingen) were performed according to the manufacturer’s instructions. Cells were stained with FITC anti-IAV Nucleoprotein antibody (Life Technologies, Waltham, MA) to determine the percentage of virally infected MoDCs. Uncompensated data were collected by analytical flow cytometry (Guava^®^ easyCyte™ 12HT flow cytometer (Luminex Corporation, Austin, TX)). Flow cytometry analysis including the percent positive cells, median fluorescence intensities (MdFI), and compensation were performed using FlowJo™ version 10.6.1 software (BD Biosciences). Compensation was performed using unstained cells for the negative population and using compensation beads for the positive fluorophore populations (Anti-Mouse Ig, κ/Negative Control Compensation Particles Set (BD Biosciences)). Fluorescence Minus One (FMO) controls were included in each biological replicate where samples were stained with all fluorophores-antibody conjugates in the panel, except the fluorophore-antibody conjugate being assessed. This corresponding sample was used to set the upper boundary for the background signal, permitting the gating of the positive population.

### T cell intracellular cytokine staining

For intracellular cytokine experiments, purified naïve T cells (Naïve Pan T Cell Isolation Kit, Miltenyi Biotec) were co-cultured alone (0:0:1) or with either mock-treated, Cal/09- or Vic/11- exposed MoDCs (4:0:1), with NK cells (0:1:1) or with both MoDCs and NK (4:1:1) for 7 days. On days 2 and 5, half of the culture medium was supplemented with fresh medium. Cells were stimulated for the last 4 h with PMA/I (eBioscience™ Cell Stimulation Cocktail) and 3 µg/mL brefeldin A and 2 mM Monensin (BioLegend^®^). During the staining process, cells were first stained with Live-or-Dye™ fixable AmCyan (Biotium, Fremont, CA) and blocked with Human TruStain FcX (Fc receptor blocking solution; BioLegend^®^) followed by fixation and permeabilization with Cytofix/Cytoperm (BD Pharmingen). Cells were stained with Pacific Blue™ anti-human CD3 (clone: UCHT1; BioLegend^®^), anti-CD4-Alexa-647 (clone: RPA-T4; BioLegend^®^), anti-CD8-phycoerythrin-Cy7 (clone: RPA-T4; BioLegend^®^), anti-human IFN-γ-phycoerythrin (PE) (clone: 4S.B3; BioLegend^®^), and anti-human TNF-FITC (clone: MAB11; BioLegend^®^) antibodies and analyzed using flow cytometry.

### RNA isolation and quantitative RT-PCR

NK cells cultured in isolation or co-cultured for 23 h with mock-treated, Cal/09- or Vic/11-exposed MoDC at a 4:1 (MoDC+NK cell) ratio were purified by MACS^®^ according to the manufacturer’s instructions (NK cell Isolation kit; Miltenyi Biotec). Total RNA from the samples was isolated using TRIzol^®^ reagent following the manufacturer’s isolation protocol (Life Technologies), by adding 5 µg of RNase-free glycogen as a carrier when precipitating RNA. RNA purity was verified by NanoDrop (Thermo Fisher Scientific) before DNase treatment and reverse transcription using SuperScript**™** IV VILO**™** Master Mix with ezDNase**™** Enzyme (Invitrogen, Waltham, MA). Primer sequences for HLA-DRA (Forward *5’-AGG CCG AGT TCT ATC TGA ATC CT-3’;* Reverse *5’-CGC CAG ACC GTC TCC TTC T-3’*) (35) and Cyclophilin (Forward *5’-TGC CAT CGC CAA GGA GTA G-3’;* Reverse *5’-TGC ACA GAC GGT CAC TCA AA-3’*) were used and HLA-DRA mRNA levels were normalized to Cyclophilin. Samples were analyzed on Bio-Rad CFX96 Touch Real-Time PCR Detection System (Bio-Rad, Hercules, CA) using Power Track SYBR Green master mix per manufacturer instructions (Thermo Fisher Scientific). The relative abundance of HLA-DRA transcript within samples was determined with the 2^−ΔΔCt^ method.

### Multiplex human cytokine magnetic bead panel

Supernatants from three donors were collected at 96 HPI and were stored at -80°C. Samples were analyzed using a custom, commercially available multiplex magnetic assay kit per manufacturer instructions (Milliplex^®^; MilliporeSigma, Burlington, MA). Briefly, supernatants harvested from cultures of T cells only; 2) MoDCs and T cells; 3) NK cells and T cells and; 4) MoDCs, NK cells, and T cells with ratios of 0:0:1, 1:0:1, 0:4:1, and 1:4:1) were transferred, in duplicates, to the provided 96-well plates to analyze cytokine concentrations. Each well received 25 μL assay buffer, followed by 25 μL of sample supernatant, and 60 μL magnetic beads. Background wells received 25 μL of assay buffer instead of 25 μL sample supernatant. Quality controls and high sensitivity human cytokine standards 1-7 were separately resuspended, inverted, and vortexed in 250 μL deionized water followed by serial dilution. The plate was sealed and incubated with shaking overnight at 4°C. After a 16 h incubation period, the plate underwent triple washes with wash buffer before adding 25 μL of detection antibodies, incubated for 1 h at room temperature, followed by adding 25 μL Streptavidin-Phycoerythrin to each well with detection antibodies. The plate was incubated for 30 min at room temperature and then washed three times. 150 μL of drive fluid was added to all wells. Magnetic beads were resuspended by using a plate shaker for five minutes. Cytokines of interest include: IFN-α2, IFN-γ, IL-2, IL-10, IL-12p70, IL-15, IL-18, TNF, and IL-21. Plates were analyzed on the MAGPIX instrument (Luminex) per manufacturer instructions. Minimum detectable concentrations for each cytokine were: IFN-α2, 2.9 pg/mL; IFN-γ, 0.8 pg/mL; IL-2, 1.0 pg/mL; IL-10, 1.1 pg/mL; IL-12p70, 0.6 pg/mL; IL-15, 1.2 pg/mL; IL-18, 0.94 pg/mL; TNF, 0.7 pg/mL; IL-21, 18.49 pg/mL. Concentrations were derived from standard curves set to a 5-parameter (log scale) curve fit.

### Statistical analysis

A Tukey’s multiple comparison test on a two-way ANOVA using GraphPad Prism V9.0.0 was performed to analyze data for transwell, and multiplex cytokine assays. Paired Wilcoxon signed-rank tests were performed using R. An alpha value of 0.05 was set to all data analysis and a p-value of < 0.05 was considered statistically significant.

### IRB approval-ethics

Blood from healthy adult donors, aged 18-65 years of age, both males and females, was used for these experiments. The research protocol for this study was approved by Midwestern University’s Institutional Review Board (IRB) under study number IRB/AZ 1299. Additional PBMCs were obtained from leukoreduction system chambers purchased from Vitalant (Scottsdale, AZ). Because these subjects were fully de-identified, the study protocol was deemed not to fall under human subjects research by the IRB at Midwestern University.

## Results

### Establishment of an autologous culture system to investigate monocyte-derived DC (MoDC)-NK cell cross-talk in response to IAV

**Figure 1:**
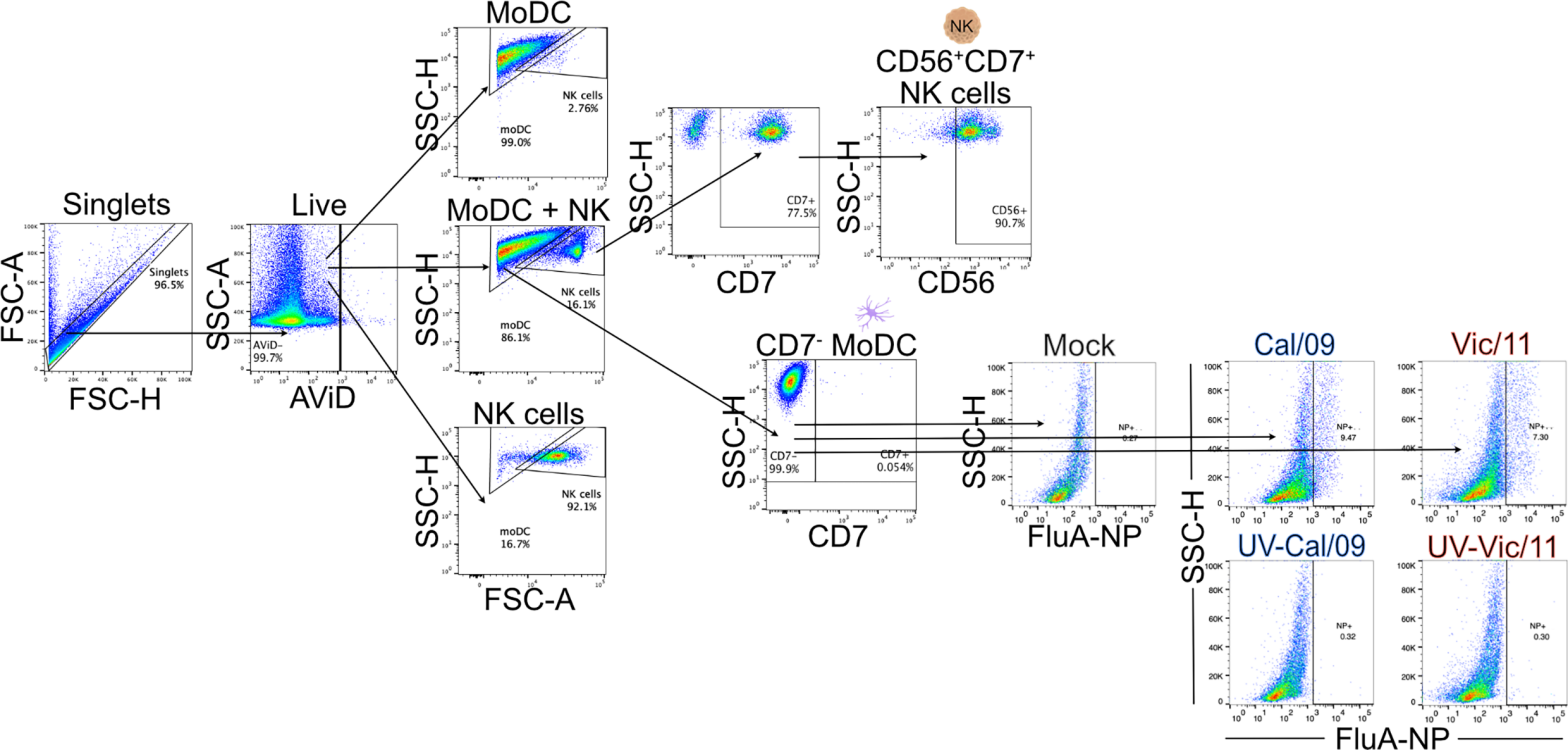
Lineage gating schematic for MoDCs and NK cells. Diagram of lineage gating tree used to identify MoDCs and NK cells by flow cytometry. MoDCs and NK cells were separated into gates by size and granularity using forward and side scatter. MoDCs were further identified as CD7^-^ while NK cells were identified as CD7^+^CD56^+^. Cal/09 and Vic/11 infection of MoDCs at an MOI of 3 at 24 HPI is shown using an antibody specific to IAV nucleoprotein (FluA-NP). AViD, LIVE/DEAD Fixable Violet Dead Cell Stain; FSC, forward scatter; SSC, side scatter.

We first established an autologous co-culture system to investigate human DC-NK cell cross-talk in response to A/California/07/2009 (H1N1) (Cal/09) and A/Victoria/361/2011 (H3N2) (Vic/11) IAV. These viruses were selected based on a prior report detailing that NK cells produced higher levels of IFN-γ in response to monocytes exposed to Cal/09 compared to Vic/11, potentially leading to distinct outcomes during DC-NK cell cross-talk (32). To generate MoDCs, monocytes were purified from cryopreserved PBMCs using negative selection followed by culturing in the presence of rhIL-4 and rhGM-CSF. After seven days, the MoDCs displayed characteristic DC morphology under the light microscope, consisting of cytoplasmic projections and the formation of loosely adherent aggregates. Expression of the monocyte marker CD14 was decreased while expression of the pathogen recognition receptor CD209 was increased when compared to freshly isolated monocytes from the same representative donor–a surface marker expression pattern consistent with MoDCs (**Figure S1A, B**) (36). The phenotypic signatures of MoDCs (CD7^-^) and NK cells (CD7^+^CD56^+^) were assessed by flow cytometry permitting lineage gating to allow for expression patterns on co-cultured MoDCs and NK cells to be analyzed individually. A representative flow cytometry gating schematic of MoDCs and NK cells is shown in **Figure 1**. MoDCs were then either mock-treated (uninfected) or exposed to Cal/09 or Vic/11 IAV strains at an MOI of 3 followed by co-culture for 23 hours (h) with autologous NK cells. Infection levels in MoDCs were tracked by staining for intracellular expression of the influenza A nucleoprotein (FluA-NP). An MOI of 3 was selected because increasing the MOI further led to a decrease in cell viability without a corresponding increase in FluA-NP^+^ cells (**Figure S1C**). At an MOI of 3, the resultant population of cells represents MoDCs that have been infected and are expressing FluA-NP and an uninfected bystander cell population with cross-priming potential. The frequency of FluA-NP^+^ MoDCs increased following exposure to both Cal/09 and Vic/11 at 24 h post-infection although not when the virus was inactivated with ultraviolet (UV) light, in dicating a subset of MoDCs were supporting *de novo* viral protein synthesis **(** **Figure 1** **)**.

### IAV decrease the frequency of CD83^+^ and CD86^+^ and increase the frequency of HLA-DR^+^ MoDCs when co-cultured with NK cells

**Figure 2:**
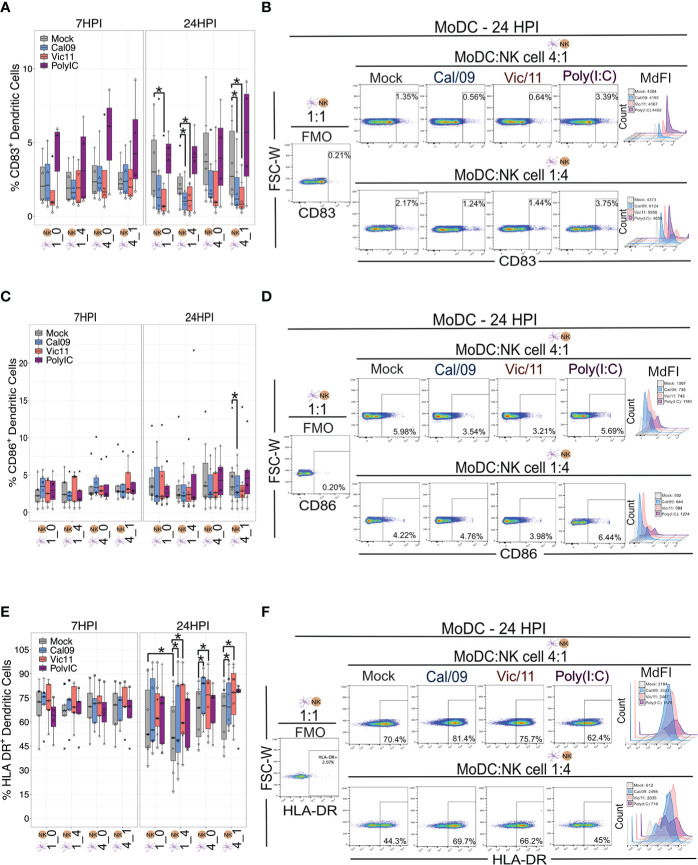
IAV exposure leads to the downregulation of CD83 and CD86 and upregulation of HLA-DR on MoDCs in co-culture with NK cells. **(A)** Summary plot of the frequency of CD83^+^ Cal/09- or Vic/11-exposed MoDCs (MOI = 3) cultured alone (1:0, 4:0) or after a 7 h (*n* = 4) or 23 h (*n* = 6) co-culture with NK cells at indicated cell ratio as assessed by flow cytometry using an antibody specific to CD83. **(B)** Representative flow plot of CD83^+^ frequency and median fluorescence intensity (MdFI) on MoDCs at a MoDC to NK cell ratio of 4:1 (top panel) or 1:4 (bottom panel) at 24 HPI (MOI = 3). **(C)** Summary plot of the frequency of CD86^+^ Cal/09- or Vic/11-exposed MoDCs (MOI = 3) cultured alone (1:0, 4:0) or after a 7 h (*n* = 5) or 23 h (*n* = 7) co-culture with NK cells at indicated cell ratio as assessed by flow cytometry using an antibody specific to CD86 **(D)** Representative flow plot of CD86^+^ frequency and MdFI on MoDCs at a MoDC to NK cell ratio of 4:1 (top panel) or 1:4 (bottom panel) at 24 HPI (MOI = 3). **(E)** Summary plot of the frequency of HLA-DR^+^ Cal/09- or Vic/11-exposed MoDCs (MOI = 3) cultured alone (1:0, 4:0) or after a 7 h (*n* = 5) or 23 h (*n* = 7) co-culture with NK cells at indicated cell ratio as assessed by flow cytometry using an antibody specific to HLA-DR. **(F)** Representative flow plot of HLA-DR^+^ frequency and MdFI on MoDCs at a MoDC to NK cell ratio of 4:1 (top panel) or 1:4 (bottom panel) at 24 HPI (MOI = 3). Poly (I:C) treatment served as a positive control. The same shape between conditions indicates that the data point is derived from the same donor. *p < 0.05, Wilcoxon signed-rank test.

Using the MoDC-NK cell co-culture model established above, we first investigated whether NK cells influenced the activation state of MoDC by measuring the expression of CD83 and CD86. These markers are DC maturation-associated phenotypic markers that are important for providing necessary co-stimulation to naïve T cells. Polyinosinic-polycytidylic acid (Poly (I:C)), a double-stranded RNA mimic recognized by Toll-like receptor 3 (TLR3) was used as a positive control to stimulate the MoDCs (37). As expected, Poly (I:C) treatment led to an increase in the frequency of CD83^+^ MoDCs, as compared to mock treatment (**Figures 2A, B**). The outcome of DC-NK cell cross-talk has been reported to vary depending on cell ratio, with high DC to NK cell ratios amplifying DC cytokine production and low DC to NK cell ratios inhibiting DC function due to killing by autologous NK cells (editing) (26, 38). Therefore, we evaluated the impact of NK cells on the expression of CD83 and CD86 at either a low (1:4) or high (4:1) DC to NK cell ratio–compared to MoDC cultured alone with equal cell numbers–1:0 or 4:0 at steady-state, uninfected conditions. The variable cell ratios explored had no significant effect on the frequency of CD83^+^ and CD86^+^ MoDCs (**Figures 2A-D**). We next evaluated whether IAV exposure affected the frequency of CD83^+^ (**Figures 2A, B**) or CD86^+^ (**Figures 2C, D**) MoDCs or the density of expression (MdFI) of CD83 and CD86. At 24 HPI, exposure of MoDCs to IAV led to lower median values of the percentage of CD83^+^ MoDCs, compared to mock-treated cells. These decreases reached statistical significance following Cal/09- and Vic/11-exposure at the low and high MoDC to NK cell ratios (1:4 and 4:1) and following Vic/11-exposure on MoDCs cultured alone (1:0) at 24 h post-infection (**Figures 2A, B**). Similar results were observed for the percentage of MoDCs expressing CD86 after a 23 h culture with NK cells at the high MoDC to NK cell ratio (4:1) when exposed to Cal/09, although not to Vic/11 (**Figures 2C, D**). Representative MdFI values of CD83 and CD86 are shown in **Figures 2B, D** and average values across all donors are compiled in **Table S1**. No statistically significant differences were reached between mock and IAV-exposed conditions within the same cell ratio. Taken together, culturing of NK cells with MoDC as the specific cell ratios examined here, failed to yield a significant impact on the percentage of CD83^+^ and CD86^+^ MoDCs compared when cultured in isolation, by contrast, exposure to IAV under a subset of conditions led to a drop in the frequency of CD83^+^ and CD86^+^ MoDCs cultured with NK cells.

We next investigated the impact of NK cells and IAV exposure on the MoDC expression of human leukocyte antigen (HLA)-DR–a subtype of HLA class II necessary for antigen presentation. Relocalization of HLA class II molecules occurs from late endosomal compartments to the plasma membrane–a process that has been shown to be increased when DCs acquire immunostimulatory properties such as expression of CD83 (39). We first evaluated whether the frequency of HLA-DR MoDC expression was influenced by the MoDC to NK cell ratios described above. Under steady-state, uninfected conditions we found that MoDCs cultured with NK cells for 23 h at a 1:4 ratio led to a decrease in the frequency of HLA-DR^+^ MoDCs compared to MoDCs cultured alone (1:0). We next tested whether IAV infection of MoDCs influenced either the percentage of HLA-DR^+^ MoDCs or the density of HLA-DR expression as assessed by MdFI. Exposure of MoDCs to Cal/09 or Vic/11 for 24 h led to an increase in the percentage of HLA-DR^+^ when cultured in isolation (4:0) or at both MoDC to NK cell ratios (1:4 or 4:1) (**Figures 2E, F**). No significant changes in the frequency of HLA-DR^+^ MoDCs were observed after Poly (I:C) treatment, consistent with results observed in prior reports (40–42). Representative MdFI values of HLA-DR are shown (**Figure 2F**) and average values across all donors are compiled in **Table S1**; no statistically significant differences were reached between mock and IAV-exposed conditions within the same cell ratio. As expected from prior reports that found that IAV infection fails to downregulate HLA class I molecules, expression of HLA class I molecules appeared similar on IAV-exposed MoDCs compared to mock treatment (**Figure S2**) (43). Together, these findings show that exposure of MoDCs to IAV increased the percentage of HLA-DR^+^ and reduced the percentage of CD83^+^ and CD86^+^ cells, consistent with a tolerogenic-like DC phenotype (44).

### IAV-exposed MoDCs induce HLA-DR expression on NK cells

**Figure 3:**
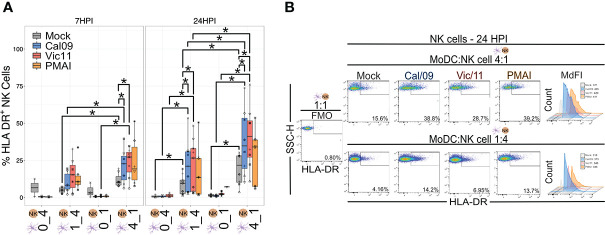
NK cells express HLA-DR after co-culture with IAV-exposed MoDCs. **(A)** Summary plot of the frequency of HLA-DR^+^ NK cells after culture alone or after 6 h (*n* = 7) or 23 h (*n* = 6-9) co-culture with mock-treated or Cal/09- or Vic/11-exposed MoDCs at an MOI of 3 as assessed by flow cytometry using an antibody specific to HLA-DR. In the NK cell-only conditions (0:1, 0:4), influenza virions were added concurrently with the NK cells. **(B)** Representative flow plot of the percentage of HLA-DR^+^ NK cells and MdFI of HLA-DR on NK cells after 23 h co-culture with Cal/09- or Vic/11-exposed MoDCs (MOI = 3) at a MoDC to NK cell ratio of 4:1 (top panel) or 1:4 (bottom panel). PMA/I treatment for 6 h served as a positive control. The same shape between conditions indicates that the data point is derived from the same donor. *p < 0.05, Wilcoxon signed-rank test.

Expression of HLA-DR is generally restricted to professional antigen-presenting cells–DCs, macrophages, and B cells–although its expression has been documented on NK cells (45). For example, an HLA-DR^+^ NK cell subset was found to be expanded in the peripheral blood of patients with primary tuberculosis and to mediate cytokine production by CD4^+^ T cells (46). We, therefore, investigated whether DC-NK cell cross-talk influenced HLA-DR expression on NK cells by evaluating the expression on NK cells cultured in isolation compared to those cultured with MoDCs. After 6 or 23 h in co-culture, there was an increase in the frequency of HLA-DR^+^ NK cells when cultured with uninfected MoDCs, with markedly higher HLA-DR expression observed at the high MoDC to NK cell ratio (4:1) and to a lesser extent at the low MoDC to NK cell ratio (1:4) (**Figures 3A, B**). Upon MoDC infection with either strain of IAV, the percentage of HLA-DR^+^ NK cells was further augmented (**Figures 3A, B**). Representative MdFI values of HLA-DR are shown in **Figure 3B** and mean values across all donors are compiled in **Table S2**. At a high MoDC to NK cell ratio (4:1), the MdFI of HLA-DR was significantly higher on Cal/09- (mean = 2185.88) and on Vic/11- (mean = 2154.25) exposed compared with uninfected MoDCs (mean = 1778.5) at 24 HPI (*p* = 0.008). In the absence of MoDCs, exposure to influenza A virions was insufficient to influence the frequency of NK cells expressing HLA-DR (**Figures 3A, B**), indicating that it is likely the exposure of NK cells to IAV-exposed MoDCs rather than to viral particles, that is responsible for the increased expression of HLA-DR on NK cells. In contrast to the increase in HLA-DR expression, NK cells failed to acquire expression of either CD83 (**Figures S3A, B**) or CD86 (**Figures S3C, D**) co-stimulatory molecules after exposure to uninfected or to IAV-exposed MoDCs. Collectively, the culture of NK cells with MoDCs leads to an increase in HLA-DR expression by NK cells, which is markedly increased when the MoDCs have been exposed to either strain of IAV.

### Direct cell-cell contact is partially required for NK cell HLA-DR expression in co-culture with IAV-exposed MoDCs

**Figure 4:**
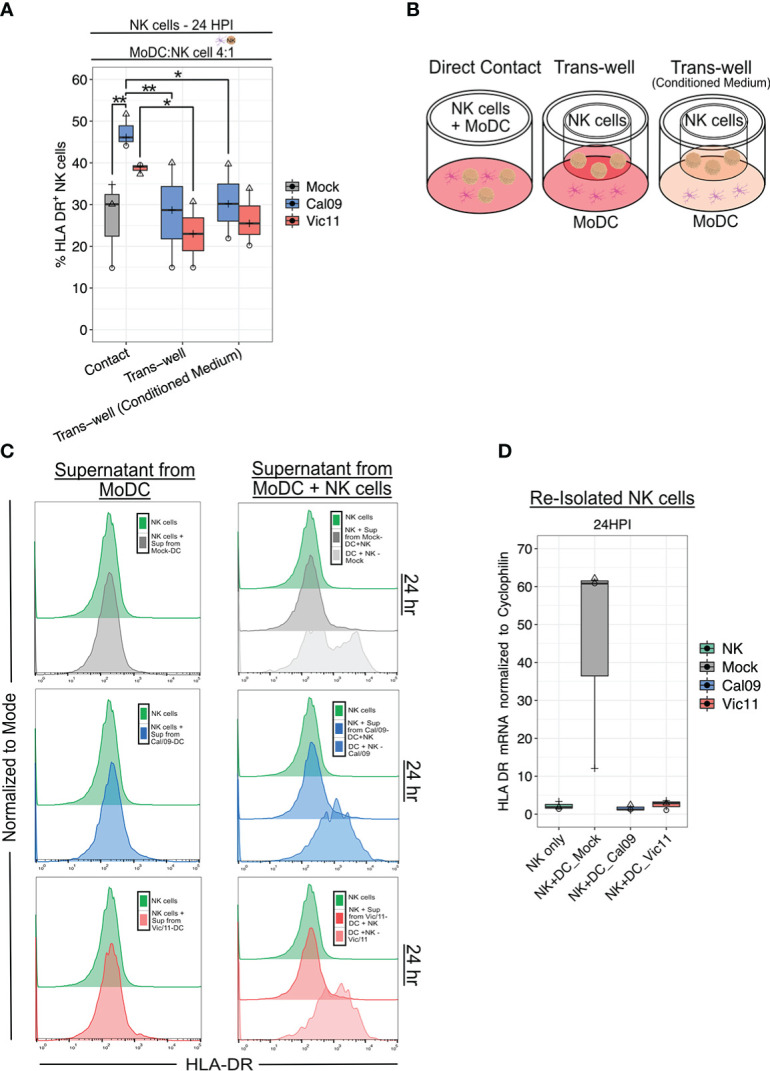
Direct contact is required for HLA-DR expression on NK cells after co-culture with IAV-exposed MoDCs. **(A)** Percentage of HLA-DR^+^ NK cells and HLA-DR MdFI after 23 h co-culture with mock-treated or IAV-exposed MoDCs at an MOI of 3 as assessed by flow cytometry using an antibody specific to HLA-DR. MoDCs were cultured in direct contact with NK cells (contact), with MoDCs seeded in the dish and NK cells seeded in the transwell with fresh medium (Trans-well) or with conditioned medium (Trans-well (conditioned medium) (*n* = 3). **(B)** Schematic of experimental set-up shown in **(A)**. **(C)** Representative flow histograms of the percentage of the maximum count of HLA-DR on NK cells after exposure to supernatant from mock-treated (top), Cal/09- (middle), or Vic/11- (bottom) exposed MoDCs cultured alone (left panel) or with NK cells (right panel) at a MoDC to NK cell ratio of 4:1 (*n =* 3). **(D)** Relative HLA-DR mRNA levels in NK cells re-purified after 23 h co-culture with mock-treated or IAV-exposed MoDCs when compared to NK cell control with RT-qPCR (*n =* 3). The same shape between conditions indicates that the data point is derived from the same donor. *p < 0.05, ns *p* > 0.05. Two-way ANOVA, Prism V9.0.0.

NK cells acquire HLA-DR either by transcriptional activation or by an intracellular membrane transfer process termed trogocytosis (47, 48). Prior work has shown that MHC class II acquisition by NK cells from DCs through trogocytosis can be abrogated by preventing cell-to-cell contact using a 0.4 µm transwell insert with a semipermeable membrane (47). We, therefore, investigated whether cell-to-cell contact was necessary for NK cell HLA-DR expression in our culture system. To this end, NK cells were co-cultured with MoDCs either in direct contact or separated by a permeable membrane (transwell). Elimination of direct cell contact led to a significant decrease in the percentage of HLA-DR^+^ NK cells when cultured with either Cal/09- or Vic/11-exposed MoDCs, however, not to baseline HLA-DR levels (**Figures 4A, B**).

We next examined the possibility that soluble mediators, such as cytokines synthesized due to receptor-ligand interactions, were also partly responsible for NK cell HLA-DR expression. To test this possibility, conditioned medium containing the soluble mediators harvested from previous IAV-exposed MoDC-NK cell 24 h co-cultures were added to MoDCs and NK cells separated by the transwell. The addition of the conditioned medium failed to rescue NK cell HLA-DR expression to the level observed with direct cell-to-cell contact (**Figures 4A, B**). Next, we asked whether cytokines produced by Cal/09- or Vic/11-exposed MoDCs either alone or in co-culture with NK cells were sufficient to induce HLA-DR NK cell expression cultured in the absence of MoDCs. Transfer of supernatant from Cal/09- or Vic/11-exposed MoDCs cultured in isolation (**Figure 4C**; left panel) or cultured with NK cells (**Figure 4C**; right panel) failed to induce the NK cell HLA-DR expression observed when cells were co-cultured (DC + NK condition; right panel). Finally, we considered whether NK cells were also transcriptionally upregulating HLA-DR in addition to acquiring it from the MoDC membrane. To this end, NK cells co-cultured with mock or IAV-exposed MoDCs were re-isolated and HLA-DR transcript levels were measured. NK cell interactions with IAV-exposed MoDCs failed to induce HLA-DR transcripts, although induction was observed in NK cells exposed to mock-treated MoDCs (**Figure 4D**). Taken together, these data are supportive of NK cells acquiring HLA-DR from the membrane of IAV-exposed DCs *via* intercellular membrane transfer, independent of the cytokine milieu.

### MoDC-NK cell cross-talk increases the frequency of CD69^+^ T cells

**Figure 5:**
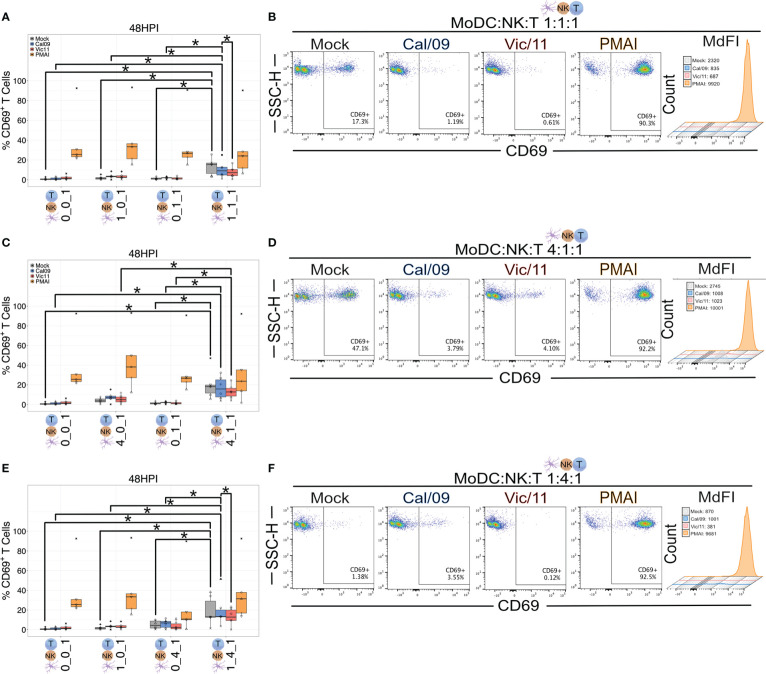
MoDC-NK cell cross-talk increases the frequency of an early marker of T cell activation. Summary plots of the frequency of CD69^+^ T cells after culture with mock-treated or Cal/09 or Vic/11-exposed MoDCs (MOI = 3) after 95 h co-culture with MoDCs and NK cells (*n* = 6) at a MoDC to NK cell to T cell ratio of **(A)** 1:1:1, **(C)** 4:1:1, and **(E)** 1:4:1 as assessed by flow cytometry using an antibody specific to CD69. Representative flow plots of the frequency of CD69^+^ T cells expression and MdFI after 47 h co-culture with Cal/09- or Vic/11-exposed MoDCs (MOI = 3) at a MoDC to NK cell to T cell ratio of **(B)** 1:1:1, **(D)** 4:1:1, and **(F)** 1:4:1. PMA/I treatment for 12 h served as a positive control. The same shape between conditions indicates that the data point is derived from the same donor. *p < 0.05, Wilcoxon signed-rank test.

We next used our co-culture system to investigate the role of MoDC-NK cell cross-talk on naïve T cell activation under steady state and IAV conditions by using flow cytometry to evaluate the surface expression of CD69. CD69 is a classic early marker of lymphocyte activation, which functions to impair T cell egress from lymph nodes–likely to promote full T cell activation (49–51). To evaluate CD69 expression, MoDCs were either mock-treated or exposed to Cal/09 or Vic/11 IAV strains followed by co-culture for 48 h with autologous NK cells and autologous naïve T cells. The 48 h time point was selected based on the finding that CD69 expression was at its peak between 18 and 48 h after anti-CD3 stimulation (52). PMA/I was used as a positive control for antigen-independent, chemical stimulation, and a representative gating schematic is shown in **Figure S4A**. To evaluate whether differing ratios of MoDC and NK cells influence T cell activation, naïve T cells were cultured in four different conditions: independently, with MoDCs, with NK cells, or in triple co-culture at MoDC: NK cell: T cell ratios of 1:1:1 (**Figures 5A, B**), 4:1:1 (**Figures 5C, D**) and 1:4:1 (**Figures 5E, F**). We first asked which condition resulted in the highest percentage of CD69^+^ T cells at steady-state, uninfected conditions. We found that the percentage of CD69^+^ T cells was highest under conditions that included both MoDCs and NK cells, compared to with MoDC alone, NK alone, or solely T cells, with the highest percentages of CD69 detected at the high MoDC to NK cell ratio (4:1) per T cell (4:1:1) (**Figures 5A-F**). We then evaluated the impact of IAV exposure of MoDCs on both the percentage of T cells expressing CD69 and the density of CD69 expression (MdFI). While the median values of the percentage of CD69^+^ T cells decreased after MoDC exposure to IAV compared to mock treatment at 1:1:1 and 4:1:1 ratios, the decrease did not reach statistical significance. Notably, at 1:1:1 and 1:4:1 ratios, the percentage of CD69^+^ T cells was significantly higher when MoDC had been exposed to Cal/09 compared to with Vic/11 (**Figures 5A, E**). Representative MdFI values of CD69 are shown (**Figures 5B, D**, **F**) and average values across all donors are compiled in **Table S3** although no statistically significant differences were reached between mock and IAV-exposed conditions within the same cell ratio. Collectively, these data support a model where early T cell activation is heightened by MoDC-NK cell cross-talk, under either steady state or certain IAV exposure conditions.

### MoDC-NK cell cross-talk increases the frequency of CD25^+^ T cells

**Figure 6:**
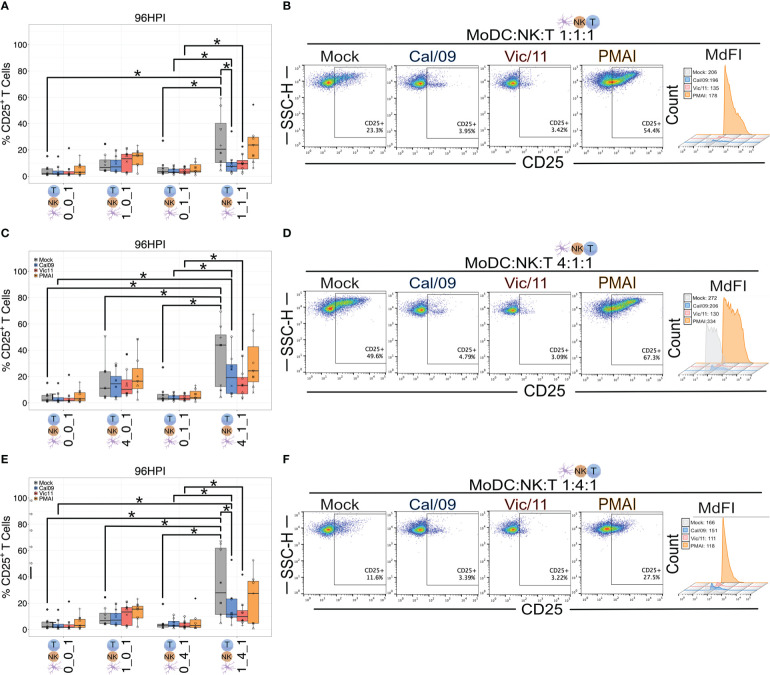
MoDC-NK cell cross-talk increases the frequency of a late marker of T cell activation. Summary plots of the frequency of CD25^+^ T cells after culture with mock-treated or Cal/09- or Vic/11-exposed MoDCs (MOI = 3) after 95 h co-culture with MoDCs and NK cells (*n* = 8) at a MoDC to NK cell to T cell ratio of **(A)** 1:1:1, **(C)** 4:1:1, and **(E)** 1:4:1 as assessed by flow cytometry using an antibody specific to CD25. Representative flow plots of the frequency of CD25^+^ T cells and MdFI after 95 h co-culture with Cal/09- or Vic/11-exposed MoDCs (MOI = 3) at a MoDC to NK cell to T cell ratio of **(B)** 1:1:1, **(D)** 4:1:1, and **(F)** 1:4:1. PMA/I treatment for 12 h served as a positive control. The same shape between conditions indicates that the data point is derived from the same donor. *p < 0.05, Wilcoxon signed-rank test.

We next assessed the impact of MoDC-NK cell cross-talk and IAV on the T cell surface expression of the alpha chain of the trimeric IL-2 receptor, CD25 (53) using the same experimental design described for CD69 and a representative gating schematic is shown in **Figure S4B**. We first asked which condition led to the highest T cell CD25 expression at steady-state, uninfected conditions. We found that similar to CD69, the frequency of T cells expressing CD25 was highest under conditions that included both MoDCs and NK cells compared to MoDC alone, NK alone, or solely T cells (**Figures 6A-F**) with the highest percentages of CD25^+^ T cells detected at the high MoDC to NK cell ratio (4:1) per T cell (4:1:1). We next evaluated the impact of MoDC IAV-exposure on both the percentage of T cells expressing CD25 and the density of CD25 expression by measuring the MdFI. IAV exposure led to a drop in median values of the percentage of CD25^+^ T cells, and these decreases reached statistical significance for Cal/09 exposure at cell ratios of 1:1:1 (**Figures 6A, B**) and 1:4:1 (**Figures 6E, F**). Representative MdFI values for CD25 expression are shown in **Figures 6B, D** and **F**, and average values across all donors are compiled in **Table S3**. The density of CD25 expression was significantly lower on T cells in triple co-culture (4:1:1) with Vic/11-exposed MoDCs (mean = 1706.5) compared with mock-treated MoDCs (mean = 2481.4) (*p* = 0.008). (**Table S3**). Taken together, these data show that MoDC-NK cell cross-talk promotes higher levels of CD25^+^ T cells, while IAV reduces the frequency of CD25^+^ T cells and the density of CD25 expression under a subset of conditions.

### IAV infection leads to lower IFN-γ, tumor necrosis factor, and IL-10 levels in MoDC-NK cell-T cell co-culture

**Figure 7:**
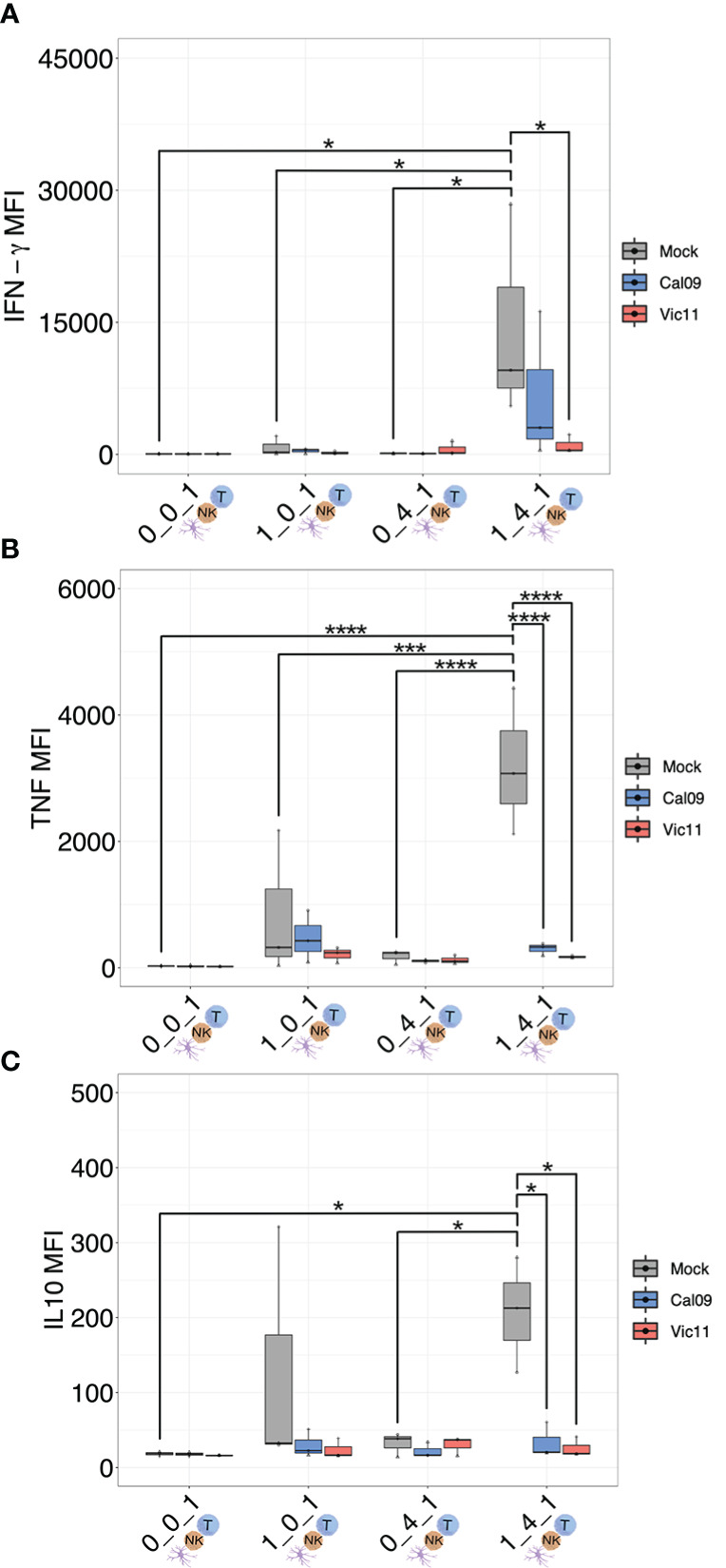
MoDC-NK cell cross-talk curtails the production of a broad array of cytokines as a result of influenza exposure. **(A)** MAGPIX data showing the mean fluorescence intensity (MFI) of **(A)** IFN-γ, **(B)** TNF, and **(C)** IL-10 present in the supernatant of either mock-treated or virion-exposed T cells (0:0:1), T cells co-cultured with mock or virus-exposed MoDCs (MOI = 3, 96 HPI) (1:0:1), T cells co-cultured with mock or virion-exposed NK cells (0:4:1) or T cells co-cultured with both virus-exposed and mock MoDCs (MOI = 3, 96 HPI) and NK cells (1:4:1) (*n* = 3). The same shape between conditions indicates that the data point is derived from the same donor. *p < 0.05, ***p < 0.0001, ****p < 0.0001. Two-way ANOVA, Prism V9.0.0.

The finding that T cell CD25 expression was augmented by co-culture with MoDCs and NK cells, yet partially abrogated by IAV, led us to ask whether the cytokine milieu differed depending on the cell types present and between mock and IAV conditions. To this end, we used a custom MAGPIX panel to measure supernatant concentrations of the cytokines IFN-α2, IFN-γ, TNF, IL-2, IL-10, IL-12p70, IL-15, IL-18, and IL-21, (**Figures 7A-C**, **Figure S5**; **Table S4**). These cytokines were selected based on their importance as key mediators of DC and NK cell cross-talk (54). T cells were either cultured in isolation (0:0:1), cultured with MoDCs (1:0:1), with NK cells (0:4:1), or with both MoDCs and NK cells (1:4:1). Influenza virions were included in 0:0:1 and 0:4:1 conditions. IFN-γ, TNF, and IL-10 were significantly increased in the 1:4:1 mock condition compared to the 0:0:1 (T cells alone) 1:0:1 (MoDCs and T cells), or 0:4:1 (NK cells and T cells) (**Figures 7A-C**). Further, the mean fluorescence intensity of IFN-γ, TNF, and IL-10 was significantly decreased in the IAV-exposed compared to uninfected samples in the 1:4:1 condition (**Figures 7A-C**). Taken together, these results show that the interaction of MoDCs with NK cells profoundly modulates the cytokine milieu when cultured with T cells, leading to increased levels of IFN-γ, TNF, and IL-10, while MoDCs exposure to IAV curtails the production of these cytokines. The precise cellular source(s) of each cytokine in the co-culture system remains unknown and could be identified in future experiments using intracellular cytokine staining paired with lineage markers for DCs, NK cells, and T cells.

### IAV infection impairs the ability of CD4^+^ and CD8^+^ T cells to secrete IFN-γ in a strain-specific manner when cultured with NK cells and MoDCs

**Figure 8:**
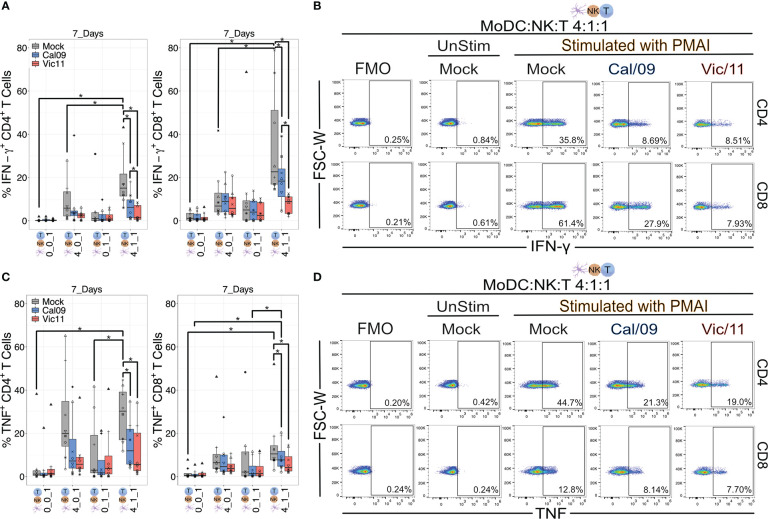
IAV-exposed MoDCs in co-culture with NK cells suppress the production of IFN-γ and TNF from CD4^+^ and CD8^+^ T cells. Summary plots of CD4^+^ and CD8^+^ T cell IFN-γ^+^ **(A)** or **(C)** TNF^+^ expression after culture with mock-treated or Cal/09- or Vic/11-exposed MoDCs (MOI = 3) after 7-day co-culture with MoDCs and NK cells at a MoDC to NK cell to T cell ratio of 0:0:1, 4:0:1, 0:1:1 or 4:1:1 followed by stimulation with PMA/I for 4 h and assessed by intracellular cytokine staining and flow cytometry using an antibody specific to IFN-γ or TNF (*n* = 7). Representative flow plots of the frequency of IFN-γ^+^ **(B)** or **(D)** TNF^+^ CD4^+^ T cells (top) and CD8^+^ T cells (bottom) after 7-day co-culture with Cal/09- or Vic/11-exposed MoDCs (MOI = 3) at a MoDC to NK cell to T cell ratio of 4:1:1 followed by stimulation with PMA/I for 4 h and assessed by flow cytometry using an antibody specific to IFN-γ or TNF. The same shape between conditions indicates that the data point is derived from the same donor. *p < 0.05, Wilcoxon signed-rank test.

IFN-γ is the major cytokine of CD4^+^ T_H_1 cells–helping to maintain the T_H_1 lineage while inhibiting differentiation into other helper T cell subsets–as well as a major effector molecule of CD8^+^ cytotoxic T cells (55–57). IFN-γ also upregulates HLA class II molecules on antigen presentation cells, thus promoting peptide-specific activation of CD4^+^ helper T cells (58). The finding that influenza infection curtailed the production of IFN-γ in the triple co-culture condition led us to investigate whether these changes impacted the ability of T cells to secrete two of the signature T_H_1 cytokines, IFN-γ and TNF (59, 60). To investigate the impact of Cal/09- or Vic/11-exposure of MoDC on the ability of helper or cytotoxic T cells to produce IFN-γ or TNF, we performed intracellular cytokine staining on CD4^+^ and CD8^+^ T cells after a 7-day co-culture with mock-treated or IAV-exposed MoDCs and NK cells. A representative lineage gating schematic is shown in **Figure S6**. 7 days was selected as this time point has been found sufficient for the generation of polarized effector subsets from human naïve T cells after MoDC co-culture (61). Naïve T cells were cultured in four different conditions: independently, with MoDCs, with NK cells, or in triple co-culture at MoDC: NK cell: T cell ratios of 4:1:1, as a high DC to NK cell ratio has been reported to amplify DC cytokine production (26, 38). PMA/I was administered for the last 4 h of co-culture to interrogate the cytokine-producing capacity of T cells under each condition. We first asked whether the presence of NK cells and MoDCs influenced T cell cytokine IFN-γ production. Indeed, the frequency of CD4^+^ and CD8^+^ T cells expressing IFN-γ was significantly higher under conditions that included both uninfected MoDCs and NK cells when compared to conditions that included only T cells, only MoDC and T cells and only NK cells and T cells, although the NK cell/T cell co-culture did not reach statistical significance (**Figure 8A**). We next asked whether exposure of MoDC to IAV impacted CD4^+^ and CD8^+^ T cell IFN-γ production. Indeed, both viruses led to an abrogated ability of CD4^+^ and CD8^+^ T cells to secrete IFN-γ in a strain-dependent manner, with Vic/11 eliciting lower IFN-γ production compared to Cal/09 (**Figures 8A, B**). In contrast, levels of T cell TNF production were not significantly higher under triple cell co-culture compared to culture with either MoDC or NK cells, although it was higher when compared to T cells cultured in isolation (**Figure 8C**). Similar to IFN-γ production, in the triple co-culture condition, exposure of MoDC to both IAV strains decreased the ability of CD4^+^ and CD8^+^ T cells to secrete TNF (**Figures 8C, D**). These results are consistent with Vic/11-infection and to a lesser extent, Cal/09-infection of MoDC in co-culture with NK cells leading to impaired ability of CD4^+^ and CD8^+^ T cells to secrete IFN-γ, the hallmark T_H_1 cytokine.

## Discussion

In this study, we found that under steady-state conditions, DC-NK cell cross-talk at varying cell ratios heightens T cell activation as indicated by an increased expression of CD69 and CD25. Consistent with these findings, multi-plex cytokine analysis on cell culture supernatants found increased levels of IL-10, TNF, and IFN-γ under MoDC-NK cell-T cell culture conditions compared to T cells cultured alone or cultured with solely MoDCs or NK cells. While the cellular source of each cytokine remains to be defined, certain NK cell subsets express Galectin-1, which upon ligation of its receptor CD69 (seen to be upregulated on T cells after a 48 h co-culture) triggers IL-10 secretion (62–64). While IL-10 is classically considered anti-inflammatory, NK cell-derived IL-10 has been shown to prevent tissue damage that occurred during murine cytomegalovirus infection (65) and to promote proliferation and cytotoxicity of human CD8^+^ T cells (66, 67). NK cells, DCs, and T cells are all capable of producing TNF and being the recipients of TNF activity, and this cytokine enhances the membrane expression of CD25 on activated T cells (68, 69). NK cells are also a major source of IFN-γ and indeed, consistent with our results, a prior study by Ferlazzo et al. found that human NK cells secrete IFN-γ in response to both immature and mature DCs under steady-state conditions (70). Importantly, NK cells are also a significant source of IFN-γ following influenza virus vaccination in humans (71) and regulate CD8^+^ T cell priming and DC migration in an IFN-γ-dependent manner in mice (72). Indeed, the ability of CD4^+^ and CD8^+^ T cells to secrete IFN-γ was highest under conditions where T cells were co-cultured with both MoDC and NK cells, compared to culture with MoDC only or to T cells cultured in isolation (**Figure 8A**).

The second focus of this study was evaluating the role of MoDC-NK cell cross-talk at varying cell ratios in regulating T cell activation in response to a pandemic and a seasonal IAV strain. We observed that IAV significantly reduced the frequency of CD25^+^ T cells when cultured with MoDCs and NK cells. This may represent a viral immune evasion strategy because a reduction in the number of high-affinity IL-2 receptors (CD25) could result in suboptimal pathogen-specific T cell expansion and memory responses (73). Indeed, other studies have demonstrated that IL-2 signaling is essential for the development of robust secondary memory CD8^+^ T cell responses during viral infection (74). Compatible with the lower levels of T cell surface markers of activation, IAV exposure reduced MoDC expression of CD83 and CD86 when cultured at a high MoDC/NK cell ratio (4:1) and curtailed the release of IL-10, TNF, and IFN-γ in the triple co-culture condition. NK cell-mediated DC maturation is dependent on TNF, therefore lower levels observed after IAV exposure could potentially hinder DC activation and subsequent stimulation of naïve T cells (26). The two IAV strains used in this study; A/California/07/2009 (Cal/09: H1N1) and A/Victoria/361/2011 (Vic/11; H3N2) have been found to exhibit qualitatively distinct responses on NK cell IFN-γ cytokine production (32), and indeed higher percentages of CD69^+^ T cells and elevated levels of the T_H_1 signature cytokine IFN-γ were detected when MoDC were exposed to Cal/09 compared to Vic/11 in triple co-culture conditions (**Figures 5A, E** and **Figure 7**). Further, exposure of MoDCs to both IAV strains curtailed the ability of CD4^+^ and CD8^+^ T cell subsets to produce IFN-γ and TNF when in triple co-culture. Of note, exposure of MoDCs to Cal/09 led to an elevated ability of helper CD4^+^ T cells to secrete the hallmark T_H_1 cytokine IFN-γ–critical for the generation of the cytotoxic T cells needed to eliminate virus-exposed cells and promote disease resolution–compared to Vic/11 (5, 75) (**Figures 8A, B**). Indeed, cytotoxic CD8^+^ T cells also displayed a markedly elevated ability to produce IFN-γ after exposure to Cal/09-exposed MoDC–compared to Vic/11, when cultured with NK cells (**Figures 8A, B**).

NK cells exhibit functional plasticity–able to switch between states including exerting their hallmark anti-viral and anti-tumor cytotoxic effector functions and, in other contexts, providing a regulatory role in dampening inflammatory immune responses (76). This functional plasticity is conferred, in part, by the *de novo* expression or upregulation of activation markers such as CD69, CD25, NKp44, CD16, and HLA-DR (77–80). Indeed, in our study, we found that IAV exposure of MoDCs followed by co-culture with NK cells led to an expansion in HLA-DR^+^ NK cells. We thus explored the mechanism responsible for the emergence of the HLA-DR^+^ NK cell subset after exposure to IAV-exposed MoDCs. We found that NK cell HLA-DR expression was reduced if direct cell-to-cell contact was prevented, consistent with prior work that found that NK cells can acquire HLA-DR from DCs through intercellular membrane transfer called “trogocytosis” (47). Prior reports have shown that cytokines, including IL-2, IL-15, IL-18, and IL-21 can promote differentiation of an HLA-DR-expressing NK cell subset (56), although the addition of supernatant from IAV-exposed MoDCs alone or in co-culture with NK cells failed to stimulate HLA-DR expression in our culture system (81). This is consistent with our multi-plex cytokine findings, which did not show significantly increased levels of these cytokines in IAV-exposed conditions compared to mock treatment (**Figure S5** and **Table S4**). Further, while HLA-DR transcripts were induced in NK cells exposed to uninfected MoDCs, they were not induced by IAV-exposed MoDCs, suggesting that the HLA-DR^+^ NK cell subset expanded by acquiring HLA-DR from the surface of IAV-exposed MoDCs.

Interestingly, prior work in a murine model found that HLA-DR^+^ NK cells can inhibit DC-induced CD4^+^ T cell responses by competitive antigen presentation, possibly due to insufficient expression of co-stimulatory molecules (47). Similarly, we found that NK cells co-cultured with IAV-exposed MoDCs failed to significantly upregulate either CD83 or CD86 co-stimulatory molecules (**Figure S3**). This raises the question of whether this human HLA-DR^+^ NK cell subset is involved in reducing IAV-mediated human T cell activation; efforts to investigate the functional role of this subset are underway. HLA-DR^+^ NK cell subsets have been found in both humans and mice and present under steady-state conditions and a range of diseases (45). For example, an HLA-DR^+^ NK cell subset was found to be expanded in the peripheral blood of patients with primary tuberculosis (46, 82). The CD56^dim^ NK cell subset from HIV-infected individuals displayed elevated HLA-DR expression and impaired IFN-γ production compared to uninfected controls (83). Here we provide the first report that culturing MoDCs with NK cells leads to an increase in human NK cell HLA-DR expression in the context of influenza infection *ex vivo*.

One potential limitation of this study is that the results were generated under *ex vivo* experimental conditions. *In vivo*, influenza viral infection may impact DC-NK cell interactions. For example, IAV infection triggers blood DC subsets to secrete chemokines (CXCL16, CXCL1, CXCL2, and CXCL3) for which NK cells have the cognate receptors (CXCR6, CXCR2) and are thus capable of potentially attracting NK cells to participate in cross-talk (84–86). Furthermore, virally infected plasmacytoid DCs produce CCL4 and CXCL10 that are capable of recruiting NK cells in chemotaxis assays (86). Isolation of PBMCs directly from healthy donors followed by isolation of autologous NK cells and differentiation of MoDCs from monocytes allows for investigation of the impact of DC and NK cell interactions in an *ex vivo* setting. Analysis of immune cells harvested from patients acutely infected with influenza, for example from bronchial lavage fluid, peripheral blood, or lymph nodes, would be of interest to evaluate whether the observed expression patterns are seen *in vivo* during an acute influenza viral infection. A second limitation is that this study chose to investigate naïve T cell responses to influenza, independent of the influenza-specific memory T population. To this end naïve T cells were isolated *via* negative selection, however, we recognize a small number of influenza-specific memory T cells may be present within the co-culture with the potential to influence the findings between PBMC donors. Finally, our system investigated only one DC subtype, which fails to recapitulate the diversity of DC subsets found *in vivo* (87). MoDCs were selected because circulating monocytes serve as a major precursor for antigen-presenting DCs within peripheral tissues including the lung (88–90). MoDCs formed *de novo* at sites of infection efficiently capture antigens, migrate to local lymph nodes, and effectively prime and cross-prime T cells to generate pathogen-specific immunity (91–93). Furthermore, they have become an attractive target for vaccine design because unlike blood DCs which comprise a very small fraction of circulating blood cells (<1%), large numbers of monocytes can be easily obtained from whole blood samples (94). Beyond the ease of collection and experimental manipulation, *in vivo* studies have also demonstrated that MoDCs are important during microbial infection and specifically in cross-priming T cells to generate pathogen-specific immunity (91, 93). Future studies to investigate the role of diverse DC subtypes are warranted and, along these lines, an allogenic plasmacytoid DC cell line has shown promising clinical observations in patients with metastatic stage IV melanoma (95).

In summary, our findings demonstrate that DC-NK cell cross-talk at the cell ratios examined herein can heighten the expression of naïve T cell surface activation markers and the ability to produce IFN-γ. We also found that IAV-exposed MoDCs after NK cell co-culture showed evidence of a tolerogenic-like activation state as determined by their decreased expression of CD83 and CD86 and increased expression of HLA-DR. Furthermore, IAV-exposed MoDCs in co-culture with NK cells and T cells led to reduced T cell activation as determined by lower surface levels of CD25 and decreased ability to secrete IFN-γ and TNF, with the seasonal 2011 Vic/11 strain triggering less IFN-γ production than the pandemic 2009 Cal/09 strain. Understanding the immune pathways that govern DC-mediated T cell responses may have future therapeutic implications. Along these lines, in the United States, the FDA has approved Sipuleucel-T, a DC-based vaccine that confers a survival benefit for patients with metastatic prostate cancer (96, 97). However, a major barrier to this type of therapeutic option includes the regulation of the DC functional state. Overall, our human *ex vivo* data may help inform the signals required to elicit an optimal DC functional state, improving the immunogenicity and efficacy of DC-based immunotherapies and vaccines. Determining whether such approaches could be leveraged to manipulate T cells to stimulate protective immunity or limit immunopathology during infection are key future endeavors.

## Data availability statement

The original contributions presented in the study are included in the article/**Supplementary Material**. Further inquiries can be directed to the corresponding author.

## Ethics statement

The studies involving human participants were reviewed and approved by Institutional Review Board, Midwestern University, Glendale, United States. The patients/participants provided their written informed consent to participate in this study.

## Author contributions

Conceptualization: LK and BL; Experiments: CU, AG, AH, JG, EP, MF, SM, SR. Analyses: LK; Writing: LK, BL. Funding: LK and BL. All authors contributed to the article and approved the submitted version.
