# Supplementary material for: Dendritic cell-natural killer cell cross-talk modulates T cell activation in response to influenza A viral infection
Source: Front Immunol. 2022 Dec 22;13:1006998. doi: 10.3389/fimmu.2022.1006998 (PMC9815106; doi:10.3389/fimmu.2022.1006998)
Supplement: Supplementary file 1 [file DataSheet_1.pdf]

Supplementary Material

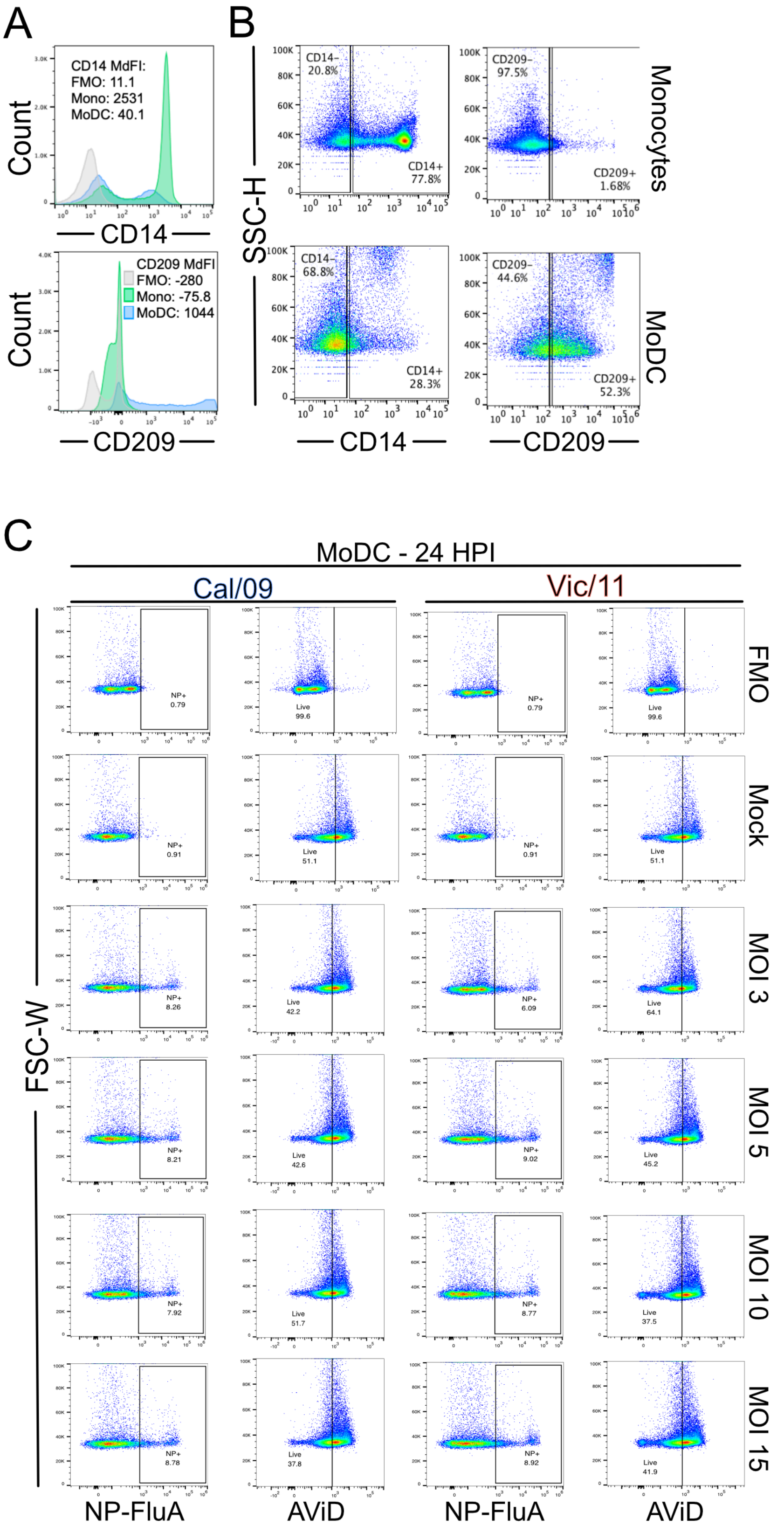

Supplementary Figures

Figure S1. Differentiation of monocytes into MoDCs. (A) Representative histograms comparing fluorescence minus one control (FMO) of CD14 (top) MdFI expression and CD209 expression (bottom) on monocytes before and after rhIL-4 and rhGM-CSF differentiation into MoDCs. **(B)** Representative flow plots of CD14 and CD209 expression on monocytes (top) before and after rhIL-4 and rhGM-CSF differentiation into MoDCs (bottom). **(C)** MoDCs were infected at the indicated MOI with either Cal/09 or Vic/11 for 24 h followed by staining with the AViD, a viability stain, and intracellular staining for influenza A nucleoprotein (FluA-NP) followed by analytical flow cytometry.

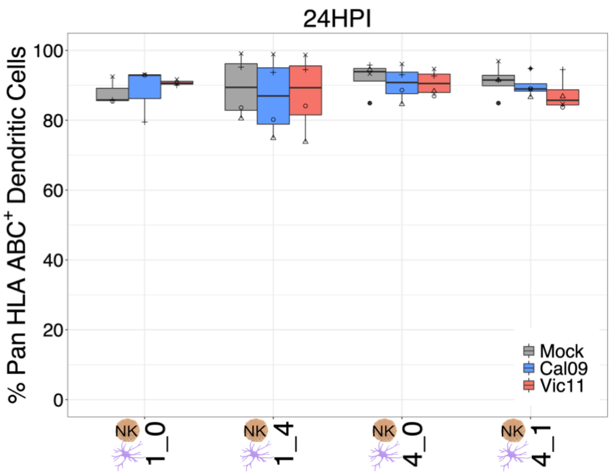

Figure S2. Expression of HLA class I molecules on MoDCs after IAV exposure. HLA Class I A, B, and C expression on MoDCs either alone (1:0 and 4:0) or after co-culture with NK cells at 1:4 or 4:1 (MoDC: NK cell) ratios for 23 h with either Cal/09- or Vic/11-exposed MoDCs at an MOI of 3 (*n* = 4).

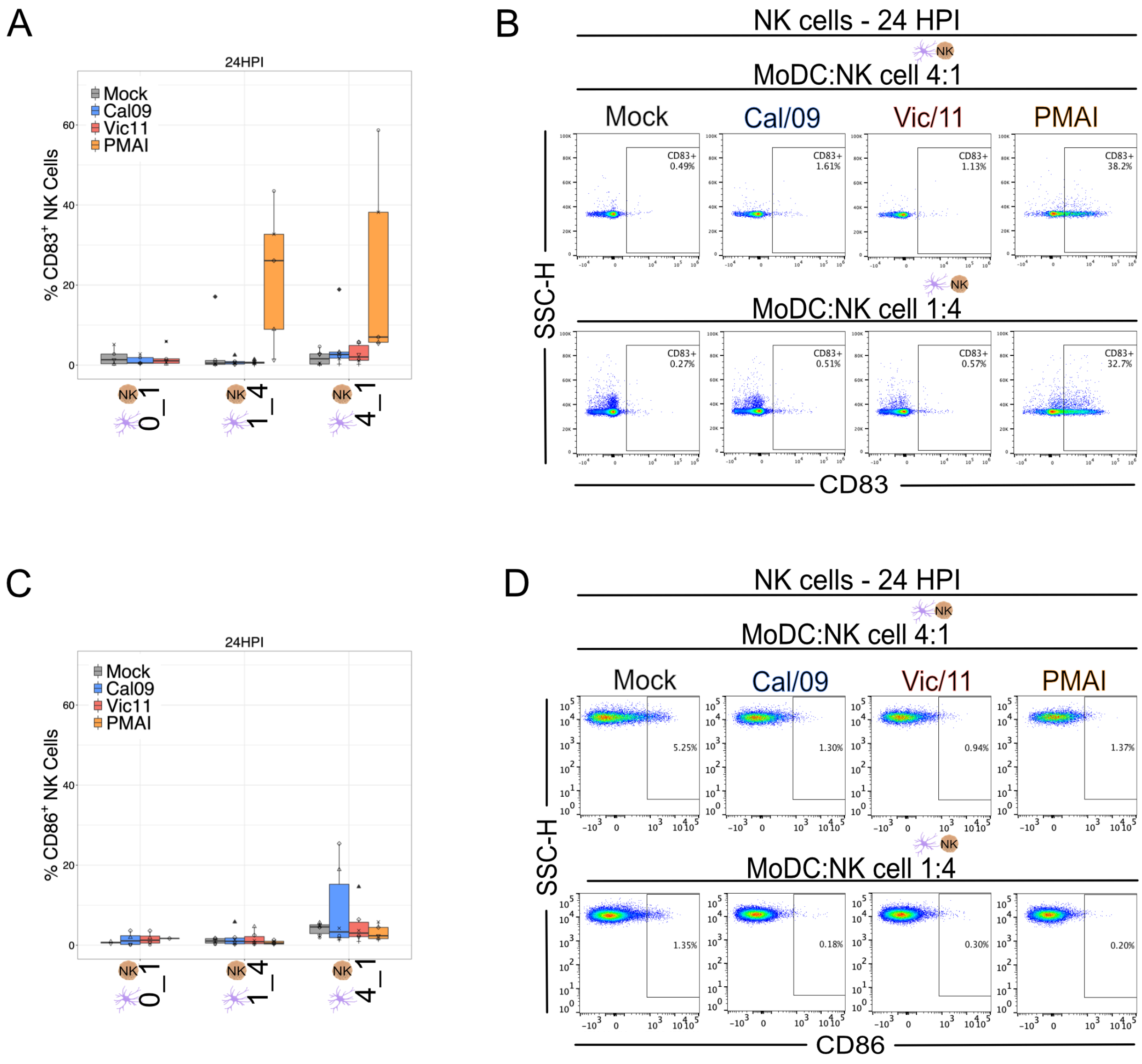

Figure S3. Expression of CD83 or CD86 on NK cells after co-culture with IAV-exposed MoDCs. Summary plot of the frequency of (**A**) CD83$^+$ or (**C**) CD86$^+$ NK cells after 23 h co-culture with mock-treated or Cal/09- or Vic/11-exposed MoDCs (MOI = 3) (*n* = 6) as assessed by flow cytometry. Representative flow plot of the percentage of CD83$^+$ (**B**) or (**D**) CD86$^+$ NK cells after 23 h co-culture with Cal/09- or Vic/11-exposed MoDCs (MOI = 3) at a MoDC to NK cell ratio of 4:1 (top panel) or 1:4 (bottom panel). PMA/I treatment for 6 h served as a positive control. The same shape between conditions indicates that the data point is derived from the same donor.

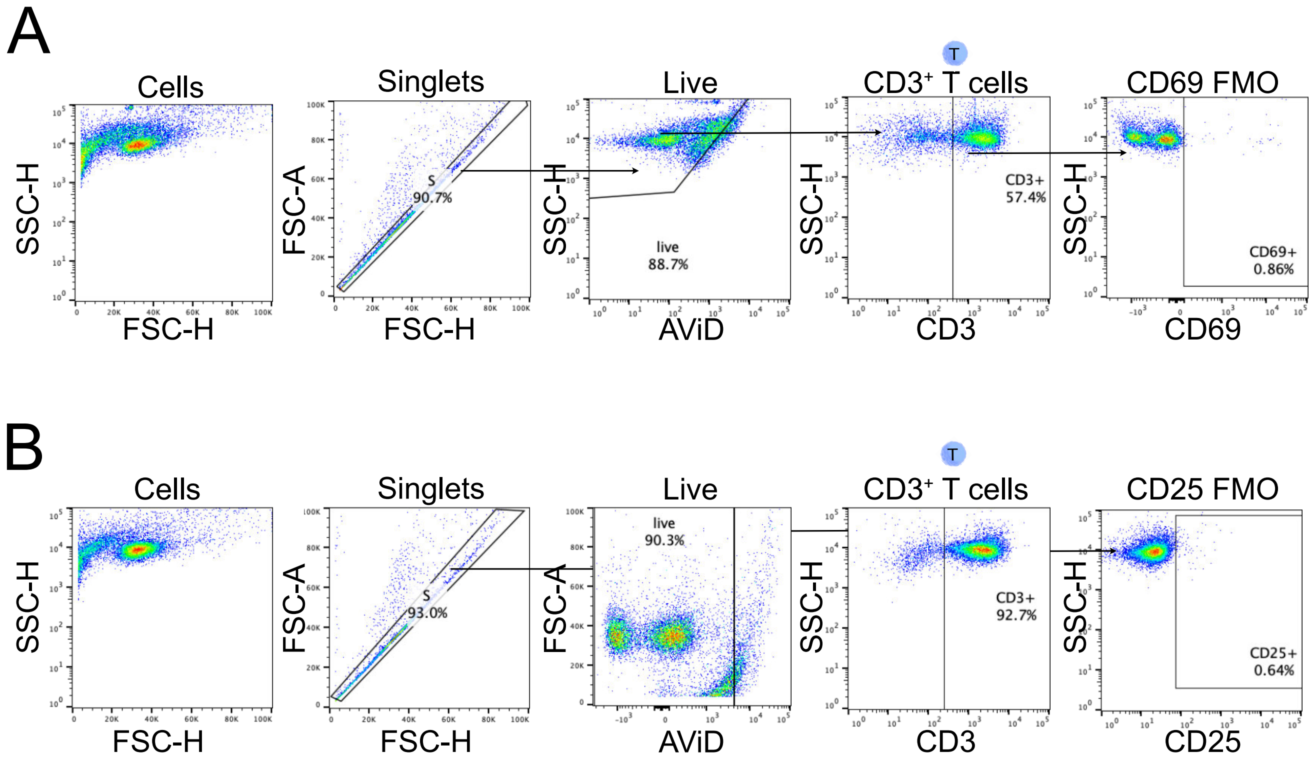

Figure S4. Lineage gating schematic for CD69⁺ and CD25⁺ T cells. Diagram of lineage gating tree used to identify **(A)** CD69⁺ and **(B)** CD25⁺ T cells using an FMO control for CD69 and CD25 to inform gating location.

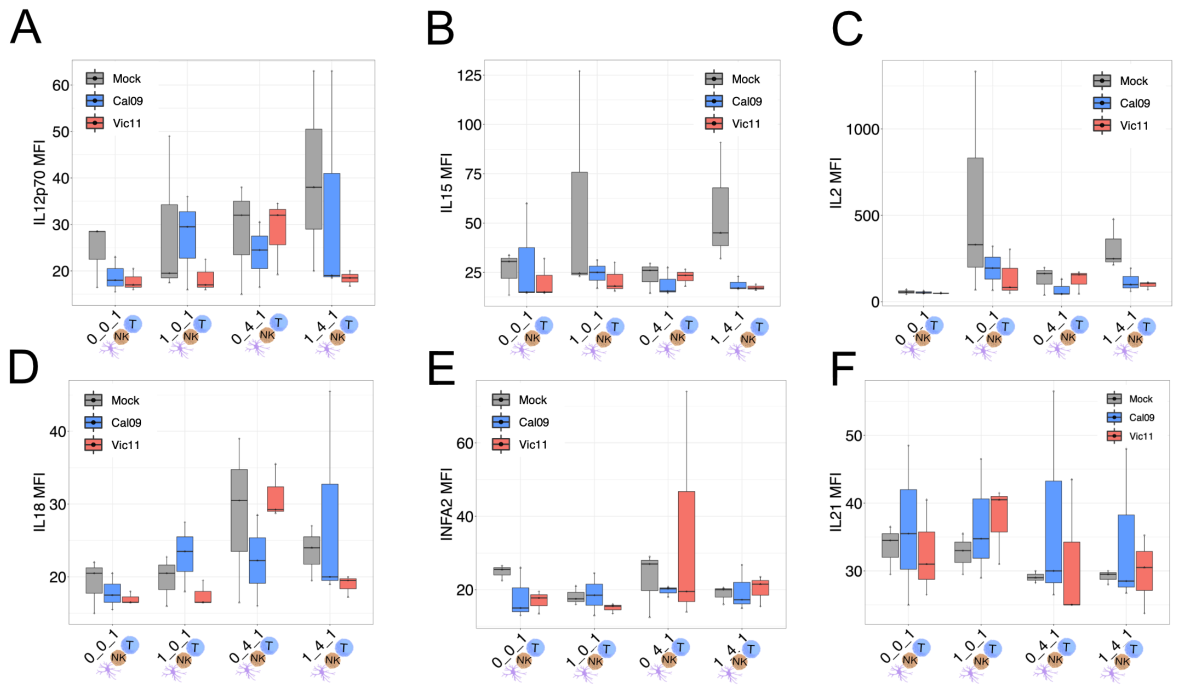

Figure S5. Quantification of six cytokines in supernatants harvested 96 h post-IAV infection from MoDC-NK cell-T cell co-cultures. MAGPIX data showing the mean fluorescence intensity (MFI) of cytokines **(A)** IL-12p70, **(B)** IL-15, **(C)** IL-2, **(D)** IL-18, **(E)** IFN-α2, **(F)** and IL-21 present in the supernatant of either mock-treated or virion-exposed T cells (0:0:1), T cell co-cultured with mock or virus-exposed MoDCs (MOI = 3, 96 HPI) (1:0:1), T cells co-cultured with mock or virion-exposed NK cells (0:4:1) or T cells co-cultured with mock or IAV-exposed MoDCs (MOI = 3, 96 HPI) and NK cells (1:4:1). The same shape between conditions indicates that the data point is derived from the same donor.

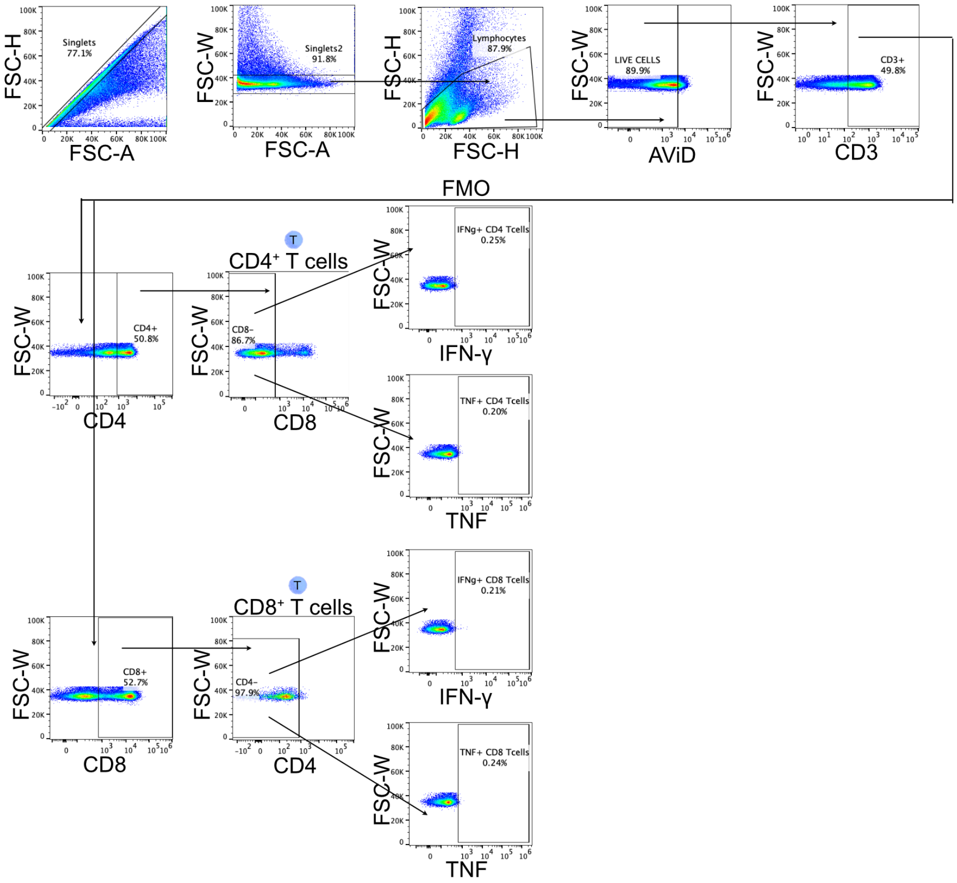

Figure S6. Lineage gating schematic for to measure the percentage of IFN-γ^+ and TNF$^+$ CD4$^+$ and CD8$^+$ T cells. Diagram of representative lineage gating tree used to identify IFN-γ^+ and TNF$^+$ CD4$^+$ T cells and IFN-γ^+ and TNF$^+$ CD8$^+$ T cells. FMO controls for IFN-γ and TNF staining on CD4$^+$ and CD8$^+$ T cells are shown.

Table S1. Mean and Standard deviation (SD) of Median Fluorescence Intensity (MdFI) of CD83, CD86, and HLA-DR on MoDCs at 24 HPI.

**MoDCs**	**CD83**	**CD86**	**HLA-DR**
Value	**Mean of MdFI ± SD**	**Mean of MdFI ± SD**	**Mean of MdFI ± SD**
**MoDC to NK cell ratio**	**1:0**	**1:0**	**1:0**
Mock	4488.666667± 3419.980624 N=6	7453.85714± 5901.79129 N=7	3407.14286 ± 1669.31757 N=7
Cal/09	4175.33333± 3111.78551 N=6	7120± 5037.04454 N=7	4115.85714± 2221.34512 N=7
Vic/11	4156± 3355.78998 N=6	8957± 8881.6435 N=7	4086.14286± 3433.71375 N=7
Poly (I:C)	2462.5± 1431.55358 N=4	5562.16667± 2708.54658 N=6	3369.2± 1549.32911 N=5
**MoDC to NK cell ratio**	**1:4**	**1:4**	**1:4**
Mock	4196.16667± 3345.14331 N=6	7327.85714± 5639.60928 N=7	3008.71429± 1525.28628 N=7
Cal/09	4487.5± 3294.48787	6491.42857± 4127.06954	3334.57143± 2193.46073

	N=6	N=7	N=7
Vic/11	4314± 3167.10473 N=6	7878.57143± 6607.48714 N=7	3887.71429± 2135.96057 N=7
Poly (I:C)	2962.75± 1736.84107 N=4	8755.33333± 7124.6789 N=6	3149.4± 1984.19361 N=5
**MoDC to NK cell ratio**	**4:0**	**4:0**	**4:0**
Mock	4098.16667± 3396.07912 N=6	6835.42857± 5024.28223 N=7	3080.57143± 1659.71512 N=7
Cal/09	4357.5± 3407.16731 N=6	6087.42857± 3578.63935 N=7	3393.85714± 1769.97066 N=7
Vic/11	4551± 3789.29972 N=6	7143.71429± 6056.16971 N=7	4085± 3622.08156 N=6
Poly (I:C)	2840.5± 1493.57122 N=4	6404.33333± 3889.87001 N=6	2676.8± 1423.77024 N=5
**MoDC to NK cell ratio**	**4:1**	**4:1**	**4:1**

Mock	4173.66667±<br>3205.49108<br>N=6	7322.28571±<br>6418.65099<br>N=7	3032.714298±<br>1825.8657<br>N=7
Cal/09	4173.33333±<br>3300.33536<br>N=6	7202±<br>5922.59071<br>N=7	3190.58±<br>2124.71304<br>N=7
Vic/11	4306±<br>3396.39727<br>N=6	8002.28571±<br>7727.8002<br>N=7	4236±<br>2858.75253<br>N=7
Poly (I:C)	3024.25±<br>1588.99557<br>N=4	6758.5±<br>5412.37729<br>N=6	2620.68±<br>1198.10655<br>N=5

Table S2. Mean and Standard deviation (SD) of Median Fluorescence Intensity (MdFI) of HLA-DR on NK cells at 24 HPI

**NK cells**	**HLA-DR**
Value	**Mean of MdFI ± SD**
**MoDC to NK cell ratio**	**0:4**
Mock	1138.667± 783.4854 N=3
Cal/09	2379± 2749.21 N=3
Vic/11	1387.25± 1753.567 N=4
**MoDC to NK cell ratio**	**1:4**
Mock	1339.11111± 1263.62885 N=9
Cal/09	1607.11111± 1403.55588 N=9
Vic/11	1633.55556± 1500.15517

	N=9
PMAI	1400.875±<br>1342.78638<br>N=8
**MoDC to NK cell ratio**	**0:1**
Mock	1927.167±<br>2277.309<br>N=6
Cal/09	1704.5±<br>1625.515<br>N=6
Vic/11	2934±<br>4497.108<br>N=6
**MoDC to NK cell ratio**	**4:1**
Mock	1778.5±<br>1684.903<br>N=8
Cal/09	2185.88±<br>1740.3<br>N=8
Vic/11	2154.25±<br>1852.395<br>N=8

PMAI	1637.875±<br>1583.49455<br>N=8

Table S3. Mean and Standard deviation (SD) of Median Fluorescence Intensity (MdFI) of CD69 (48 HPI) and CD25 (96 HPI) on $CD3^+$ naïve T cells

**T cells**	**CD69**	**CD25**
Value	**Mean of MdFI ± SD**	**Mean of MdFI ± SD**
**MoDC to NK cell to T cell ratio**	0:0:1	0:0:1
Mock	1623±<br>2067.69176<br>N=6	1426±<br>1906.48442<br>N=8
Cal/09	1998.5±<br>2383.79997<br>N=6	1321.75±<br>1723.44603<br>N=8
Vic/11	1734.33333±<br>1948.94378<br>N=6	1377.625±<br>1794.65412<br>N=8
PMAI	4447±<br>4041.43199<br>N=6	660.285714±<br>421.636777<br>N=7
**MoDC to NK cell to T cell ratio**	1:0:1	1:0:1

Mock	1625.33333±<br>2266.66492<br>N=6	1593.125±<br>1954.05296<br>N=8
Cal/09	1368.66667±<br>2126.17343<br>N=6	1779.625±<br>2389.62603<br>N=8
Vic/11	696.8±<br>620.771053<br>N=5	1590.75±<br>1769.26536<br>N=8
PMAI	5205.6±<br>4558.75606<br>N=6	903.857143±<br>686.645573<br>N=7
**MoDC to NK cell to T cell ratio**	0:1:1	0:1:1
Mock	1847.66667±<br>2085.58842<br>N=6	1432.375±<br>1789.63555<br>N=8
Cal/09	1551.66667±<br>2222.09835<br>N=6	1563.125±<br>1991.05316<br>N=8
Vic/11	1507±<br>2296.56282<br>N=6	1463.75±<br>1611.72869<br>N=8
PMAI	4280.4±	837.714286±

	3402.43329<br>N=6	611.11775<br>N=7
**MoDC to NK cell to T cell ratio**	1:1:1	1:1:1
Mock	2003.66667±<br>2344.95328<br>N=6	1623.75±<br>1834.37594<br>N=8
Cal/09	1963.5±<br>2995.95619<br>N=6	2653.875±<br>4947.84604<br>N=8
Vic/11	1863±<br>2825.55644<br>N=6	1338.125±<br>1617.06122<br>N=8
PMAI	4856.2±<br>4530.7142<br>N=6	1225±<br>1095.50323<br>N=7
**MoDC to NK cell to T cell ratio**	4:0:1	4:0:1
Mock	616.2±<br>510.780481<br>N=5	1381.75±<br>1360.93915<br>N=8
Cal/09	1538.83333±<br>2099.08775<br>N=6	1870.75±<br>2672.58894<br>N=8
Vic/11	1462.33333±	1660.25±

	2168.47436<br>N=6	1727.90672<br>N=8
PMAI	5026.2±<br>3716.91958<br>N=6	964.428571±<br>587.338022<br>N=7
**MoDC to NK cell to T cell ratio**	4:1:1	4:1:1
Mock	1979.83333±<br>2376.43173<br>N=6	2481.375±<br>3514.3585<br>N=8
Cal/09	2126.16667±<br>3279.17315<br>N=6	2631.75±<br>4438.26693<br>N=8
Vic/11	1897.5±<br>2772.54199<br>N=6	1706.5±<br>2488.04731<br>N=8
PMAI	4139.4±<br>3704.34695<br>N=6	1402.71429±<br>1173.03619<br>N=7
**MoDC to NK cell to T cell ratio**	0:4:1	0:4:1
Mock	1799.66667±<br>2287.47124<br>N=6	1507.25±<br>1782.62613<br>N=8
Cal/09	1948.5±	1777.375±

	2343.73409<br>N=6	2074.90936<br>N=8
Vic/11	1543.16667±<br>2187.4379<br>N=6	1566.125±<br>1855.29316<br>N=8
PMAI	4086.8±<br>4032.57978<br>N=6	886.428571±<br>687.374924<br>N=7
**MoDC to NK cell to T cell ratio**	1:4:1	1:4:1
Mock	2187.16667±<br>2903.74134<br>N=6	3000.625±<br>4369.05946<br>N=8
Cal/09	2153±<br>3097.51559<br>N=6	3034.93±<br>5459.55996<br>N=8
Vic/11	2006.33333±<br>2937.00621<br>N=6	1973.125±<br>2683.17336<br>N=8
PMAI	4515.6±<br>3487.32101<br>N=6	1559.57143±<br>1636.4164<br>N=7

Table S4. Raw MFI values of cytokines in supernatants harvested 96 h post-IAV infection from MoDC-NK cell-T cell co-cultures quantified by MAGPIX.

Donor	Treatment	Condition	Cytokine	Value (MFI)
HIP1	Mock	1_4_1	IFNA2	16
HIP1	Mock	1_0_1	IFNA2	17.5
HIP1	Mock	0_4_1	IFNA2	29
HIP1	Mock	0_0_1	IFNA2	22.5
HIP1	Cal09	1_4_1	IFNA2	26.75
HIP1	Cal09	1_0_1	IFNA2	24.5
HIP1	Cal09	0_4_1	IFNA2	18
HIP1	Cal09	0_0_1	IFNA2	15
HIP1	Vic11	1_4_1	IFNA2	23.5
HIP1	Vic11	1_0_1	IFNA2	15.5
HIP1	Vic11	0_4_1	IFNA2	19.5
HIP1	Vic11	0_0_1	IFNA2	17.75
HIP9	Mock	1_4_1	IFNA2	20.5
HIP9	Mock	1_0_1	IFNA2	21
HIP9	Mock	0_4_1	IFNA2	12.5
HIP9	Mock	0_0_1	IFNA2	25.5

HIP9	Cal09	1_4_1	IFNA2	15
HIP9	Cal09	1_0_1	IFNA2	13
HIP9	Cal09	0_4_1	IFNA2	20.75
HIP9	Cal09	0_0_1	IFNA2	26
HIP9	Vic11	1_4_1	IFNA2	21.5
HIP9	Vic11	1_0_1	IFNA2	16
HIP9	Vic11	0_4_1	IFNA2	14
HIP9	Vic11	0_0_1	IFNA2	13.5
HIP18	Mock	1_4_1	IFNA2	20
HIP18	Mock	1_0_1	IFNA2	16
HIP18	Mock	0_4_1	IFNA2	27
HIP18	Mock	0_0_1	IFNA2	26.5
HIP18	Cal09	1_4_1	IFNA2	17.25
HIP18	Cal09	1_0_1	IFNA2	18.5
HIP18	Cal09	0_4_1	IFNA2	20.25
HIP18	Cal09	0_0_1	IFNA2	13
HIP18	Vic11	1_4_1	IFNA2	15.5

HIP18	Vic11	1_0_1	IFNA2	13.5
HIP18	Vic11	0_4_1	IFNA2	74
HIP18	Vic11	0_0_1	IFNA2	19.5
HIP1	Mock	1_4_1	IFNG	9560
HIP1	Mock	1_0_1	IFNG	229.5
HIP1	Mock	0_4_1	IFNG	143.5
HIP1	Mock	0_0_1	IFNG	47
HIP1	Cal09	1_4_1	IFNG	3030
HIP1	Cal09	1_0_1	IFNG	518
HIP1	Cal09	0_4_1	IFNG	65.5
HIP1	Cal09	0_0_1	IFNG	48
HIP1	Vic11	1_4_1	IFNG	444.5
HIP1	Vic11	1_0_1	IFNG	381
HIP1	Vic11	0_4_1	IFNG	149
HIP1	Vic11	0_0_1	IFNG	45
HIP9	Mock	1_4_1	IFNG	28414
HIP9	Mock	1_0_1	IFNG	44

HIP9	Mock	0_4_1	IFNG	43
HIP9	Mock	0_0_1	IFNG	58.5
HIP9	Cal09	1_4_1	IFNG	450
HIP9	Cal09	1_0_1	IFNG	52
HIP9	Cal09	0_4_1	IFNG	89.5
HIP9	Cal09	0_0_1	IFNG	42
HIP9	Vic11	1_4_1	IFNG	438.5
HIP9	Vic11	1_0_1	IFNG	106
HIP9	Vic11	0_4_1	IFNG	1516
HIP9	Vic11	0_0_1	IFNG	47
HIP18	Mock	1_4_1	IFNG	5516
HIP18	Mock	1_0_1	IFNG	2078
HIP18	Mock	0_4_1	IFNG	120
HIP18	Mock	0_0_1	IFNG	41
HIP18	Cal09	1_4_1	IFNG	16225
HIP18	Cal09	1_0_1	IFNG	621
HIP18	Cal09	0_4_1	IFNG	100

HIP18	Cal09	0_0_1	IFNG	43
HIP18	Vic11	1_4_1	IFNG	2247
HIP18	Vic11	1_0_1	IFNG	81
HIP18	Vic11	0_4_1	IFNG	106
HIP18	Vic11	0_0_1	IFNG	47
HIP1	Mock	1_4_1	IL2	212.5
HIP1	Mock	1_0_1	IL2	1333
HIP1	Mock	0_4_1	IL2	197.5
HIP1	Mock	0_0_1	IL2	55.5
HIP1	Cal09	1_4_1	IL2	193.5
HIP1	Cal09	1_0_1	IL2	320.5
HIP1	Cal09	0_4_1	IL2	44.75
HIP1	Cal09	0_0_1	IL2	51.5
HIP1	Vic11	1_4_1	IL2	107
HIP1	Vic11	1_0_1	IL2	303.5
HIP1	Vic11	0_4_1	IL2	170.5
HIP1	Vic11	0_0_1	IL2	47.75

HIP9	Mock	1_4_1	IL2	477.75
HIP9	Mock	1_0_1	IL2	69.25
HIP9	Mock	0_4_1	IL2	39.25
HIP9	Mock	0_0_1	IL2	70.5
HIP9	Cal09	1_4_1	IL2	98.75
HIP9	Cal09	1_0_1	IL2	66.75
HIP9	Cal09	0_4_1	IL2	131
HIP9	Cal09	0_0_1	IL2	62.5
HIP9	Vic11	1_4_1	IL2	111.5
HIP9	Vic11	1_0_1	IL2	83
HIP9	Vic11	0_4_1	IL2	46.5
HIP9	Vic11	0_0_1	IL2	51.75
HIP18	Mock	1_4_1	IL2	248.5
HIP18	Mock	1_0_1	IL2	330
HIP18	Mock	0_4_1	IL2	163
HIP18	Mock	0_0_1	IL2	43.5
HIP18	Cal09	1_4_1	IL2	60

HIP18	Cal09	1_0_1	IL2	194.5
HIP18	Cal09	0_4_1	IL2	44
HIP18	Cal09	0_0_1	IL2	45.25
HIP18	Vic11	1_4_1	IL2	70
HIP18	Vic11	1_0_1	IL2	49.5
HIP18	Vic11	0_4_1	IL2	156
HIP18	Vic11	0_0_1	IL2	48.5
HIP1	Mock	1_4_1	IL10	126.75
HIP1	Mock	1_0_1	IL10	30
HIP1	Mock	0_4_1	IL10	44
HIP1	Mock	0_0_1	IL10	19
HIP1	Cal09	1_4_1	IL10	20.5
HIP1	Cal09	1_0_1	IL10	22.5
HIP1	Cal09	0_4_1	IL10	16.5
HIP1	Cal09	0_0_1	IL10	18
HIP1	Vic11	1_4_1	IL10	18.5
HIP1	Vic11	1_0_1	IL10	16.5

HIP1	Vic11	0_4_1	IL10	38
HIP1	Vic11	0_0_1	IL10	17
HIP9	Mock	1_4_1	IL10	280.5
HIP9	Mock	1_0_1	IL10	32.5
HIP9	Mock	0_4_1	IL10	14
HIP9	Mock	0_0_1	IL10	21
HIP9	Cal09	1_4_1	IL10	19.25
HIP9	Cal09	1_0_1	IL10	16.5
HIP9	Cal09	0_4_1	IL10	34
HIP9	Cal09	0_0_1	IL10	20.5
HIP9	Vic11	1_4_1	IL10	17.75
HIP9	Vic11	1_0_1	IL10	15.5
HIP9	Vic11	0_4_1	IL10	15.5
HIP9	Vic11	0_0_1	IL10	16
HIP18	Mock	1_4_1	IL10	212.5
HIP18	Mock	1_0_1	IL10	321
HIP18	Mock	0_4_1	IL10	38.5

HIP18	Mock	0_0_1	IL10	15
HIP18	Cal09	1_4_1	IL10	60.5
HIP18	Cal09	1_0_1	IL10	51
HIP18	Cal09	0_4_1	IL10	16
HIP18	Cal09	0_0_1	IL10	15
HIP18	Vic11	1_4_1	IL10	41
HIP18	Vic11	1_0_1	IL10	39
HIP18	Vic11	0_4_1	IL10	37
HIP18	Vic11	0_0_1	IL10	16
HIP1	Mock	1_4_1	IL12p70	20
HIP1	Mock	1_0_1	IL12p70	19.5
HIP1	Mock	0_4_1	IL12p70	38
HIP1	Mock	0_0_1	IL12p70	28.5
HIP1	Cal09	1_4_1	IL12p70	18.5
HIP1	Cal09	1_0_1	IL12p70	29.5
HIP1	Cal09	0_4_1	IL12p70	16.5
HIP1	Cal09	0_0_1	IL12p70	18

HIP1	Vic11	1_4_1	IL12p70	18.5
HIP1	Vic11	1_0_1	IL12p70	17
HIP1	Vic11	0_4_1	IL12p70	32
HIP1	Vic11	0_0_1	IL12p70	20.5
HIP9	Mock	1_4_1	IL12p70	38
HIP9	Mock	1_0_1	IL12p70	17.5
HIP9	Mock	0_4_1	IL12p70	15
HIP9	Mock	0_0_1	IL12p70	28.5
HIP9	Cal09	1_4_1	IL12p70	19
HIP9	Cal09	1_0_1	IL12p70	16
HIP9	Cal09	0_4_1	IL12p70	30.5
HIP9	Cal09	0_0_1	IL12p70	23
HIP9	Vic11	1_4_1	IL12p70	16.75
HIP9	Vic11	1_0_1	IL12p70	16
HIP9	Vic11	0_4_1	IL12p70	19.25
HIP9	Vic11	0_0_1	IL12p70	17
HIP18	Mock	1_4_1	IL12p70	63

HIP18	Mock	1_0_1	IL12p70	49
HIP18	Mock	0_4_1	IL12p70	32
HIP18	Mock	0_0_1	IL12p70	16.5
HIP18	Cal09	1_4_1	IL12p70	63
HIP18	Cal09	1_0_1	IL12p70	36
HIP18	Cal09	0_4_1	IL12p70	24.5
HIP18	Cal09	0_0_1	IL12p70	15.5
HIP18	Vic11	1_4_1	IL12p70	20
HIP18	Vic11	1_0_1	IL12p70	22.5
HIP18	Vic11	0_4_1	IL12p70	34.5
HIP18	Vic11	0_0_1	IL12p70	16
HIP1	Mock	1_4_1	IL15	32
HIP1	Mock	1_0_1	IL15	23
HIP1	Mock	0_4_1	IL15	29.5
HIP1	Mock	0_0_1	IL15	30.5
HIP1	Cal09	1_4_1	IL15	16.5
HIP1	Cal09	1_0_1	IL15	31.25

HIP1	Cal09	0_4_1	IL15	14.5
HIP1	Cal09	0_0_1	IL15	15
HIP1	Vic11	1_4_1	IL15	17
HIP1	Vic11	1_0_1	IL15	15.5
HIP1	Vic11	0_4_1	IL15	26.5
HIP1	Vic11	0_0_1	IL15	32
HIP9	Mock	1_4_1	IL15	45
HIP9	Mock	1_0_1	IL15	24.5
HIP9	Mock	0_4_1	IL15	14.5
HIP9	Mock	0_0_1	IL15	33.75
HIP9	Cal09	1_4_1	IL15	17
HIP9	Cal09	1_0_1	IL15	17
HIP9	Cal09	0_4_1	IL15	27.5
HIP9	Cal09	0_0_1	IL15	60
HIP9	Vic11	1_4_1	IL15	16
HIP9	Vic11	1_0_1	IL15	18
HIP9	Vic11	0_4_1	IL15	18

HIP9	Vic11	0_0_1	IL15	15
HIP18	Mock	1_4_1	IL15	90.75
HIP18	Mock	1_0_1	IL15	127
HIP18	Mock	0_4_1	IL15	26
HIP18	Mock	0_0_1	IL15	13.5
HIP18	Cal09	1_4_1	IL15	23
HIP18	Cal09	1_0_1	IL15	25
HIP18	Cal09	0_4_1	IL15	15.5
HIP18	Cal09	0_0_1	IL15	14.5
HIP18	Vic11	1_4_1	IL15	19
HIP18	Vic11	1_0_1	IL15	30
HIP18	Vic11	0_4_1	IL15	23.5
HIP18	Vic11	0_0_1	IL15	14.5
HIP1	Mock	1_4_1	IL18	19.5
HIP1	Mock	1_0_1	IL18	20.5
HIP1	Mock	0_4_1	IL18	39
HIP1	Mock	0_0_1	IL18	20.5

HIP1	Cal09	1_4_1	IL18	20
HIP1	Cal09	1_0_1	IL18	23.5
HIP1	Cal09	0_4_1	IL18	22.25
HIP1	Cal09	0_0_1	IL18	17.5
HIP1	Vic11	1_4_1	IL18	20
HIP1	Vic11	1_0_1	IL18	16.5
HIP1	Vic11	0_4_1	IL18	28.75
HIP1	Vic11	0_0_1	IL18	18
HIP9	Mock	1_4_1	IL18	24
HIP9	Mock	1_0_1	IL18	16
HIP9	Mock	0_4_1	IL18	16.5
HIP9	Mock	0_0_1	IL18	22
HIP9	Cal09	1_4_1	IL18	19
HIP9	Cal09	1_0_1	IL18	18
HIP9	Cal09	0_4_1	IL18	28.5
HIP9	Cal09	0_0_1	IL18	20.5
HIP9	Vic11	1_4_1	IL18	17.25

HIP9	Vic11	1_0_1	IL18	16.5
HIP9	Vic11	0_4_1	IL18	35.5
HIP9	Vic11	0_0_1	IL18	16.5
HIP18	Mock	1_4_1	IL18	27
HIP18	Mock	1_0_1	IL18	22.75
HIP18	Mock	0_4_1	IL18	30.5
HIP18	Mock	0_0_1	IL18	15
HIP18	Cal09	1_4_1	IL18	45.5
HIP18	Cal09	1_0_1	IL18	27.5
HIP18	Cal09	0_4_1	IL18	16
HIP18	Cal09	0_0_1	IL18	15.5
HIP18	Vic11	1_4_1	IL18	19.5
HIP18	Vic11	1_0_1	IL18	19.5
HIP18	Vic11	0_4_1	IL18	29.25
HIP18	Vic11	0_0_1	IL18	16.5
HIP1	Mock	1_4_1	TNF	3074.5
HIP1	Mock	1_0_1	TNF	322

HIP1	Mock	0_4_1	TNF	236
HIP1	Mock	0_0_1	TNF	27.5
HIP1	Cal09	1_4_1	TNF	381.75
HIP1	Cal09	1_0_1	TNF	908
HIP1	Cal09	0_4_1	TNF	77.5
HIP1	Cal09	0_0_1	TNF	20
HIP1	Vic11	1_4_1	TNF	196.75
HIP1	Vic11	1_0_1	TNF	316.5
HIP1	Vic11	0_4_1	TNF	201.5
HIP1	Vic11	0_0_1	TNF	22
HIP9	Mock	1_4_1	TNF	4424
HIP9	Mock	1_0_1	TNF	33.5
HIP9	Mock	0_4_1	TNF	50.75
HIP9	Mock	0_0_1	TNF	33.25
HIP9	Cal09	1_4_1	TNF	191.5
HIP9	Cal09	1_0_1	TNF	88
HIP9	Cal09	0_4_1	TNF	124.25

HIP9	Cal09	0_0_1	TNF	41.75
HIP9	Vic11	1_4_1	TNF	154.5
HIP9	Vic11	1_0_1	TNF	73
HIP9	Vic11	0_4_1	TNF	66
HIP9	Vic11	0_0_1	TNF	18.25
HIP18	Mock	1_4_1	TNF	2118
HIP18	Mock	1_0_1	TNF	2171.5
HIP18	Mock	0_4_1	TNF	256.25
HIP18	Mock	0_0_1	TNF	17
HIP18	Cal09	1_4_1	TNF	327.25
HIP18	Cal09	1_0_1	TNF	427.5
HIP18	Cal09	0_4_1	TNF	116
HIP18	Cal09	0_0_1	TNF	15.5
HIP18	Vic11	1_4_1	TNF	168.25
HIP18	Vic11	1_0_1	TNF	238.75
HIP18	Vic11	0_4_1	TNF	103.75
HIP18	Vic11	0_0_1	TNF	19.5

HIP1	Mock	1_4_1	IL21	28
HIP1	Mock	1_0_1	IL21	33
HIP1	Mock	0_4_1	IL21	30
HIP1	Mock	0_0_1	IL21	34.5
HIP1	Cal09	1_4_1	IL21	48
HIP1	Cal09	1_0_1	IL21	46.5
HIP1	Cal09	0_4_1	IL21	56.5
HIP1	Cal09	0_0_1	IL21	48.5
HIP1	Vic11	1_4_1	IL21	35.25
HIP1	Vic11	1_0_1	IL21	31
HIP1	Vic11	0_4_1	IL21	25
HIP1	Vic11	0_0_1	IL21	31
HIP9	Mock	1_4_1	IL21	29.5
HIP9	Mock	1_0_1	IL21	35.5
HIP9	Mock	0_4_1	IL21	29
HIP9	Mock	0_0_1	IL21	36.5
HIP9	Cal09	1_4_1	IL21	26.75

HIP9	Cal09	1_0_1	IL21	29
HIP9	Cal09	0_4_1	IL21	26.5
HIP9	Cal09	0_0_1	IL21	25
HIP9	Vic11	1_4_1	IL21	30.5
HIP9	Vic11	1_0_1	IL21	40.5
HIP9	Vic11	0_4_1	IL21	43.5
HIP9	Vic11	0_0_1	IL21	40.5
HIP18	Mock	1_4_1	IL21	30
HIP18	Mock	1_0_1	IL21	29.5
HIP18	Mock	0_4_1	IL21	28.25
HIP18	Mock	0_0_1	IL21	29.5
HIP18	Cal09	1_4_1	IL21	28.5
HIP18	Cal09	1_0_1	IL21	34.75
HIP18	Cal09	0_4_1	IL21	30
HIP18	Cal09	0_0_1	IL21	35.5
HIP18	Vic11	1_4_1	IL21	23.75
HIP18	Vic11	1_0_1	IL21	41.5

HIP18	Vic11	0_4_1	IL21	25
HIP18	Vic11	0_0_1	IL21	26.5
